# Risk of Cardiovascular Diseases Associated With Medications Used in Attention-Deficit/Hyperactivity Disorder: A Systematic Review and Meta-analysis

**DOI:** 10.1001/jamanetworkopen.2022.43597

**Published:** 2022-11-23

**Authors:** Le Zhang, Honghui Yao, Lin Li, Ebba Du Rietz, Pontus Andell, Miguel Garcia-Argibay, Brian M. D’Onofrio, Samuele Cortese, Henrik Larsson, Zheng Chang

**Affiliations:** 1 Department of Medical Epidemiology and Biostatistics, Karolinska Institute, Sweden; 2 School of Medical Sciences, Örebro University, Örebro, Sweden; 3 Unit of Cardiology, Department of Medicine, Karolinska Institutet, Stockholm, Sweden; 4 Heart and Vascular Division, Karolinska University Hospital, Stockholm, Sweden; 5 Department of Psychological and Brain Sciences, Indiana University, Bloomington; 6 Centre for Innovation in Mental Health-Developmental Lab, School of Psychology, University of Southampton and NHS Trust, Southampton, United Kingdom; 7 Hassenfeld Children’s Hospital at NYU Langone, New York University Child Study Center, New York; 8 Division of Psychiatry and Applied Psychology, School of Medicine, University of Nottingham, Nottingham, United Kingdom

## Abstract

**Importance:**

Use of attention-deficit/hyperactivity disorder (ADHD) medications has increased substantially over the past decades, but there are concerns regarding their cardiovascular safety.

**Objective:**

To provide an updated synthesis of evidence on whether ADHD medications are associated with the risk of a broad range of cardiovascular diseases (CVDs).

**Data Sources:**

PubMed, Embase, PsycINFO, and Web of Science up to May 1, 2022.

**Study Selection:**

Observational studies investigating the association between ADHD medications (including stimulants and nonstimulants) and risk of CVD.

**Data Extraction and Synthesis:**

Independent reviewers extracted data and assessed study quality using the Good Research for Comparative Effectiveness (GRACE) checklist. Data were pooled using random-effects models. This study is reported according to the Meta-analyses of Observational Studies in Epidemiology guideline.

**Main Outcomes and Measures:**

The outcome was any type of cardiovascular event, including hypertension, ischemic heart disease, cerebrovascular disease, heart failure, venous thromboembolism, tachyarrhythmias, and cardiac arrest.

**Results:**

Nineteen studies (with 3 931 532 participants including children, adolescents, and adults; 60.9% male), of which 14 were cohort studies, from 6 countries or regions were included in the meta-analysis. Median follow-up time ranged from 0.25 to 9.5 years (median, 1.5 years). Pooled adjusted relative risk (RR) did not show a statistically significant association between ADHD medication use and any CVD among children and adolescents (RR, 1.18; 95% CI, 0.91-1.53), young or middle-aged adults (RR, 1.04; 95% CI, 0.43-2.48), or older adults (RR, 1.59; 95% CI, 0.62-4.05). No significant associations for stimulants (RR, 1.24; 95% CI, 0.84-1.83) or nonstimulants (RR, 1.22; 95% CI, 0.25-5.97) were observed. For specific cardiovascular outcomes, no statistically significant association was found in relation to cardiac arrest or arrhythmias (RR, 1.60; 95% CI, 0.94-2.72), cerebrovascular diseases (RR, 0.91; 95% CI, 0.72-1.15), or myocardial infarction (RR, 1.06; 95% CI, 0.68-1.65). There was no associations with any CVD in female patients (RR, 1.88; 95% CI, 0.43-8.24) and in those with preexisting CVD (RR, 1.31; 95% CI, 0.80-2.16). Heterogeneity between studies was high and significant except for the analysis on cerebrovascular diseases.

**Conclusions and Relevance:**

This meta-analysis suggests no statistically significant association between ADHD medications and the risk of CVD across age groups, although a modest risk increase could not be ruled out, especially for the risk of cardiac arrest or tachyarrhythmias. Further investigation is warranted for the cardiovascular risk in female patients and patients with preexisting CVD as well as long-term risks associated with ADHD medication use.

## Key Points

**Question:**

Are attention-deficit/hyperactivity disorder (ADHD) medications associated with the risk of cardiovascular disease (CVD)?

**Findings:**

This systematic review and meta-analysis based on 19 observational studies with more than 3.9 million participants suggested that there was no statistically significant association between ADHD medications and the risk of cardiovascular events among children and adolescents, young and middle-aged adults, or older adults.

**Meaning:**

Despite no statistically significant association between ADHD medications and CVD, more evidence is needed for the potential risk of cardiac arrest and tachyarrhythmias, the cardiovascular risk in female patients and in those with preexisting CVD, and long-term risk.

## Teaser

This systematic review and meta-analysis provides an updated synthesis of evidence on whether attention-deficit/hyperactivity disorder (ADHD) medications are associated with the risk of a broad range of cardiovascular diseases (CVDs).

## Introduction

Attention-deficit/hyperactivity disorder (ADHD), one of the most common neurodevelopmental disorders, is characterized by developmentally inappropriate inattention and/or hyperactivity-impulsivity symptoms starting in childhood.^1^ The symptoms often persist into adulthood,^2,3^ and even into older age for a substantial number of patients.^4^ ADHD medications, including both stimulants and nonstimulants, are recommended for pharmacological treatment of ADHD, and the prevalence of ADHD medication use among both children and adults has increased substantially in many countries.^5^

While evidence from randomized clinical trials (RCTs) suggests ADHD medications are efficacious in reducing core ADHD symptoms,^6^ there are concerns about their cardiovascular safety.^7^ As ADHD medications are sympathomimetic agents that exert dopaminergic and noradrenergic effects, increasing heart rate and blood pressure is biologically plausible.^8^ A previous Cochrane review of RCTs found that the stimulant methylphenidate was associated with increased pulse or heart rate.^7^ However, as these RCTs could only evaluate short-term effects, it remains uncertain whether these changes led to a clinically significant risk of cardiovascular disease (CVD) over time. Longitudinal observation studies evaluating serious cardiovascular outcomes associated with ADHD medication use have emerged during the last decade, but with mixed findings.^9,10,11,12^ A review paper incorporating five large population-based studies in the US reported no association between stimulants and serious cardiovascular events in children.^13^ A meta-analysis of only 3 studies^14^ found no increased risk of arrhythmic and ischemic cardiac events but a decreased risk of stroke. A more recent meta-analysis of 10 studies^15^ showed a positive association between ADHD medications and risk of sudden death or arrhythmia but not for stroke, myocardial infarction, or all-cause mortality. However, it had several methodology limitations (eg, not preregistered, narrow outcome definition, and missing several important studies). Moreover, several new original studies have been published after these meta-analyses.^16,17,18,19,20^ Thus, an updated synthesis is needed to address those limitations as well as to include a broader range of cardiovascular events (eg, hypertension, heart failure, and transient ischemic attack that have not been included in previous meta-analyses) and conduct sub-analyses by type of cardiovascular events and ADHD medications. In addition, observational studies that evaluate the benefits or risks of medical treatments are prone to bias (eg, immortal time bias, prevalent user bias, confounding by indication) if not conducted appropriately. It is therefore critical to make a rigorous quality assessment of the available studies and discuss common problems that future studies need to address. Understanding whether, and to what extent, ADHD medications are associated with CVD is highly relevant from both clinical and public health perspectives, as an increasing number of individuals are receiving ADHD medications globally. Findings of any significant association would prompt research on underlying causal mechanisms (eg, dopaminergic dysfunction and alterations in cytochrome P450 2D6 metabolism).^8,21^

The current study aims to provide a comprehensive and updated systematic review and meta-analysis to assess the associations between ADHD medications and risks of a broad range of cardiovascular events. In addition, we aim to examine whether there is any difference in the associations by types of ADHD medication, types of cardiovascular events, sex, age, and preexisting CVD conditions.

## Methods

This study was conducted and reported according to the Meta-analyses of Observational Studies in Epidemiology (MOOSE) checklist.^22^ Our protocol is registered in the International Prospective Register of Systematic Reviews (CRD42021283702).^23^

### Search Strategy and Selection of Studies

A systematic search for observational studies was conducted in MEDLINE via PubMed, Embase, PsycINFO, and Web of Science, up to May 1, 2022. We used various combinations of the following keywords: *cardiovascular disease*, *coronary heart disease*, *heart disease*, *sudden death*, *ischemic heart disease*, *hypertension*, *cerebrovascular disease*, *stroke*, *transient ischemic attack*, *attention-deficit hyperactivity disorder*, *central nervous system stimulants*, and *observational study*. No restrictions to language were applied. The search strategy was designed with the assistance of a university librarian at Karolinska Institute (eTable 1 in the Supplement). In addition, we performed manual searches through the reference lists of relevant original publications and reviews to identify further pertinent studies.

We included all types of observational studies investigating associations between ADHD medication use and the risk of any CVD. We excluded reports, review articles, animal research, RCTs, and conference abstracts; studies without a comparator group; and studies with abuse or misuse of ADHD medication as the exposure. Titles, abstracts, and full text of included studies were screened independently by 2 investigators (L.Z. and H.Y.). Discrepancies were resolved through discussion with a senior investigator (L.L.).

### Data Extraction

The following information was extracted from each study for the qualitative and quantitative synthesis: first author, year of publication, sample size, data source, study country, age and sex distribution, study design, year of original data collection, follow-up time, type of ADHD medication, measure of medication use, definition of CVD, relative risk, and covariate adjustment. Two investigators conducted the data extraction separately (L.Z. and H.Y.), and any disagreements were resolved through discussion with a senior investigator (L.L.).

Good Research for Comparative Effectiveness (GRACE) checklist version 2 was used for quality assessment.^24^ Unlike the commonly used Newcastle-Ottawa Scale,^25^ the GRACE checklist is tailored for evaluating the quality of observational studies that examine the outcomes of medical treatment. It evaluates the quality of observational research based on the use of concurrent comparators, equivalent measurement of outcomes in different groups, collection of data on confounders and effect modifiers, risk of immortal time bias, and reporting of sensitivity analysis.^24^ Eleven items in the GRACE Checklist are grouped into 2 groups reflecting the quality of data and methods (eTable 2 in the Supplement). The quality assessment was completed by two investigators independently (L.Z. and H.Y.), and any discrepancies were solved by discussing with a senior investigator (L.L.).

### Statistical Analysis

The characteristics of all included studies were described. Hazard ratios (HRs) from Cox regression, incidence rate ratios (IRRs) from Poisson regression, and odds ratios (ORs) from logistic regression were combined as approximations to relative risks (RRs), because under rare event assumption, different effect measures would yield mathematically similar estimates.^26,27^ We used random-effects models to account for heterogeneity between studies. The significance of heterogeneity across studies was examined using Cochran *Q* test, while the percentage of variation attributed to true heterogeneity was estimated using the inconsistency index (*I*^2^).^28^ The restricted maximum likelihood method was used to estimate between-study variability, with the Hartung-Knapp-Sidik-Jonkman confidence interval for the summary estimates.^29,30^

We meta-analyzed adjusted RRs across all studies and by age groups (children and adolescents, young and middle-aged adults, and older adults). To evaluate each study’s influence on the pooled estimates, the leave-one-out analysis was conducted. Publication bias was first assessed through visual inspection of the funnel plot and then tested quantitatively with Egger test. Subgroup analyses were conducted to investigate the associations of (1) stimulant and nonstimulant medications with any CVD, (2) ADHD medications with specific CVD (ie, cardiac arrest or tachyarrhythmias, cerebrovascular disease, myocardial infarction), (3) stimulant ADHD medications with specific CVD, (4) ADHD medications with any CVD in individuals with and without a history of CVD, and (5) ADHD medications with any CVD by sex. All analyses were performed with Stata version 16.0 (StataCorp). Statistical significance was set at *P* < .05, and all tests were 2-tailed.

## Results

### Study Characteristics

**Figure 1.**
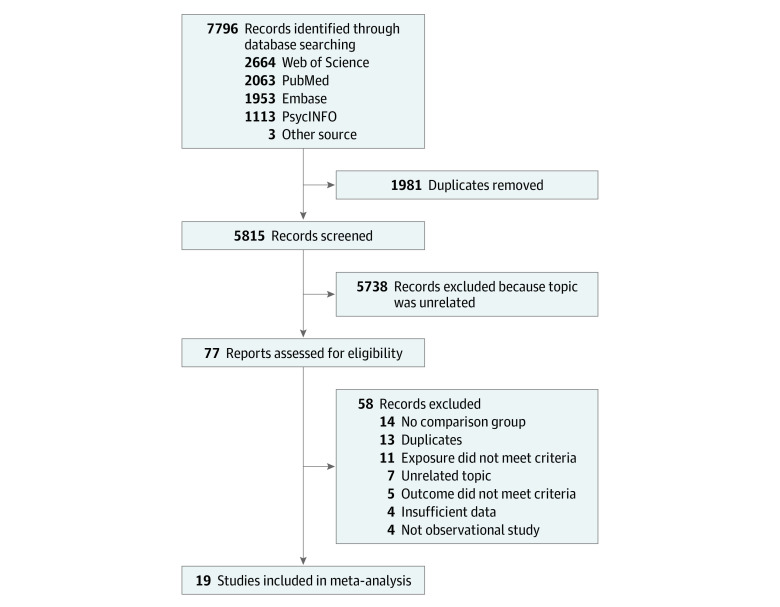
Flow Diagram for Inclusion of Studies

The process of study selection is shown in Figure 1. Detailed information on excluded articles with reasons is shown in eTable 3 in the Supplement. Overall, we included 19 studies published during 2007 to 2021, and their main characteristics are presented in Table 1.^9,10,11,12,16,17,18,19,20,31,32,33,34,35,36,37,38,39,40^ A total of 3 931 532 participants from 6 countries or regions (United States, South Korea, Canada, Denmark, Spain, and Hong Kong) were included. The study samples included children, adolescents, and adults, and 60.9% of participants were male. Average follow-up time ranged from 0.25 to 9.5 (median, 1.5) years. Most studies (14) were cohort studies,^9,10,11,12,18,20,32,33,34,35,36,37,39,40^ followed by 3 nested case-control studies,^16,19,31^ and 2 self-controlled case series.^17,38^ The most common data source was insurance claims databases (15 studies).^9,10,12,16,17,20,31,32,33,35,36,37,38,39^ More than half of the studies (10) used incident users,^17,20,31,32,33,35,36,37,38,39^ while others included prevalent users.^9,10,11,12,16,18,19,34,40^ Most studies used *International Classification of Diseases, Ninth Revision *or *International Statistical Classification of Diseases and Related Health Problems, Tenth Revision * to define CVD, except 1 study^34^ that used self-reported CVD and another^40^ without sufficient information on outcome measurement. Absolute risks of CVD in the included studies are shown in eTable 4 in the Supplement. All studies adjusted for measured covariates as an attempt to control for confounding, but the included covariates varied substantially across studies. The 2 self-controlled case series studies^17,38^ further accounted for unmeasured confounders that are time invariant. The GRACE quality scores ranged from 5 to 11 (median, 9). Immortal time bias and lack of meaningful sensitivity analyses were the most common limitations in studies with lower scores (eTable 5 in the Supplement).

**Table 1.** Characteristics of Included Studies

Source (country)	Data source	Year of data collection	Study design	Median follow-up, y	Participants, No.	Male, %	Age, y	Exposure				Outcomes		Adjustment for covariates	GRACE score
								ADHD medication	New or prevalent user	Measure of use	Duration of use, y	Definition	Diseases		
Cooper et al,^9^ 2011 (United States)	Claims	1986-2005	Cohort	2.1	1 200 438	71	Mean, 11; range, 2-24	AMP, MPH, DMPH, ATX, pemoline	Mixed^a^	Current	NA	*ICD-9*, *ICD-10*	MI, CA, stroke	Demographics, comorbidities, health care utilization	11
Dalsgaard et al,^11^ 2014 (Denmark)	Register	1990-2008	Cohort	9.5	8300	81	Mean, 11	AMP, MPH	Mixed^a^	Current	2.5	*ICD-10*	CVD	Demographics, comorbidities, perinatal characteristics	8
Guertin et al,^31^ 2014 (Canada)	Claims	2001-2010	NCC	NA	38 495	70	Mean, 9	AMP, MPH, ATX	New	Current	NA	*ICD-9*, *ICD-10*	CVD	Demographics	8
Habel et al,^10^ 2011 (United States)	Claims	1986-2005	Cohort	1.3	443 198	46	Range, 25-64	AMP, MPH, ATX, pemoline	Mixed^a^	Current	0.33	*ICD-9*, *ICD-10*	MI, CA, stroke	Demographics, CV risk score	11
Holick et al,^32^ 2009 (United States)	Claims	2003-2006	Cohort	1.5	86 205	52	≥18	AMP, MPH, ATX	New	Current	NA	*ICD-9*	Cerebrovascular disease, TIA	Demographics, comorbidities, comedications	9
Houghton et al,^16^ 2020 (United States)	Claims	2000-2016	NCC	NA	2046	68	Mean, 14; range, 3-18	AMP, MPH, ATX	Mixed^a^	Current	NA	*ICD-9*, *ICD-10*	MI, arrhythmia, stroke	Demographics, CV risk, comorbidities, comedications	9
Jeong et al,^17^ 2021 (South Korea)	Claims	2002-2018	SCCS	NA	2104	51	Mean, 58; ≥6	MPH	New	Current	2.8	*ICD-10*	MI	Age, comorbidities, comedications	10
Jeong et al,^17^ 2021 (Taiwan)	Claims	2004-2015	SCCS	NA	484	64	Mean, 65; ≥6	MPH	New	Current	2.3	*ICD-9*	MI	Age, comorbidities, comedications	10
Jeong et al,^17^ 2021 (Hong Kong)	Clinical data	2001-2016	SCCS	NA	30	50	Mean, 70; ≥48	MPH	New	Current	2.3	*ICD-9*	MI	Age, comorbidities, comedications	10
Latronica et al,^18^ 2021 (United States)	EHR	2018-2020	Cohort	2	13 233	37	Mean, 70; ≥65	AMP	Mixed^a^	Current	NA	*ICD-10*	HF, stroke, MI, AF, IHD, arrhythmia	Demographics, BMI, comorbidities	7
Olfson et al,^33^ 2012 (United States)	Claims	1997-2007	Cohort	1.8	171 126	67	Range, 6-21	AMP, MPH	New	Current	NA	*ICD-9*	Angina pectoris, arrhythmia, TIA	Demographics, comorbidities, comedications	9
Peyre et al,^34^ 2014 (United States)	National representative survey	2004-2005	Cohort	1	807	59	Mean, 40	Not specified	Mixed^a^	Ever	NA	Self-report	MI, angina pectoris, stroke	CV risk factors	5
Saiz et al,^19^ 2020 (Spain)	Primary care database	2002-2014	NCC	NA	2882	40	Mean, 14; range, 5-25	MPH	Mixed^a^	Current	0.65	ICPC, *ICD-9*	Valvular heart disease	Age, sex, smoking, comorbidities, comedications	9
Schelleman et al,^35^ 2011 (United States)	Claims	1999-2006	Cohort	1.4	241 417	72	Range, 3-17	AMP, MPH, ATX	New	Current	0.37	*ICD-9*	Sudden death, ventricular arrhythmia	Data source	8
Schelleman et al,^36^ 2012 (United States)	Claims	1999-2006	Cohort	1.2	219 954	45	≥18	MPH	New	Current	0.16	*ICD-9*	MI, stroke	Demographics, comorbidities, comedications	9
Schelleman et al,^37^ 2013 (United States)	Claims	1999-2006	Cohort	1.2	192 905	46	≥18	AMP	New	Current	0.24	*ICD-9*	MI, stroke	Demographics, comorbidities, comedications	9
Shin et al,^38^ 2016 (South Korea)	Claims	2008-2011	SCCS	NA	1224	78	Mean, 13; range, ≤17	MPH	New	Current	0.5	*ICD-10*	Arrythmias, HF, Hypertension, MI, stroke	Age, comorbidities, comedications	11
Tadrous et al,^20^ 2021 (Canada)	Claims and health care database	2002-2016	Cohort	1	31 310	49	Mean, 74; range, ≥66	AMP, MPH	New	Current	NA	*ICD-9*, *ICD-10*	MI, ventricular arrhythmia, stroke, TIA	Demographics, CVD history, physician visits, comorbidities, comedications	11
Winterstein et al,^39^ 2007 (United States)	Claims	1994-2004	Cohort	2.3	55 383	70	Range, 3-20	AMP, MPH, pemoline	New	Current	NA	*ICD-9*	MI, hypertensive diseases, aortic or thoracic aneurysm, arrhythmia, cardiac symptoms	Age, race, congenital anomalies, history of circulatory disease, comorbidities, comedications	9
Winterstein et al,^12^ 2012 (United States)	Claims	1999-2006	Cohort	1.9	1 219 847	59	Range, 3-18	AMP, MPH	Mixed^a^	Current	NA	*ICD-9*	MI, CA, ventricular arrhythmia, stroke	Demographics, CV risk factors, comorbidities, and comedications	9
Zhang et al,^40^ 2015 (United States)	Research sample	1979-2014	Cohort	7.9	144	62	Mean, 11	AMP, MPH, ATX, guanfacine	Mixed^a^	Current	NA	NA	Syncope, aborted CA	Age, gender, QTc-duration, prior cardiac event, comedications	6

Abbreviations: ADHD, attention-deficit/hyperactivity disorder; AF, atrial fibrillation; AMP, amphetamines; ATX, atomoxetine; BMI, body mass index; CA, cardiac arrest; CV, cardiovascular; CVD, cardiovascular disease; DMPH, dexmethylphenidate; EHR, electronic health record; HF, heart failure; EHR, electronic health records; GRACE, The Good Research for Comparative Effectiveness Checklist; *ICD-9*, *International Classification of Diseases, Ninth Revision*;* ICD-10*,* International Statistical Classification of Diseases and Related Health Problems, Tenth Revision*; ICPC, international classification of primary care; IHD, ischemic heart disease; MI, myocardial infarction; MPH, methylphenidate; NA, not available; NCC, nested case-control; SCCS, self-controlled case series; TIA; transient ischemic attack.

^a^ Mixed indicates mixture of incident and prevalent users.

### Meta-analysis Results

**Figure 2.**
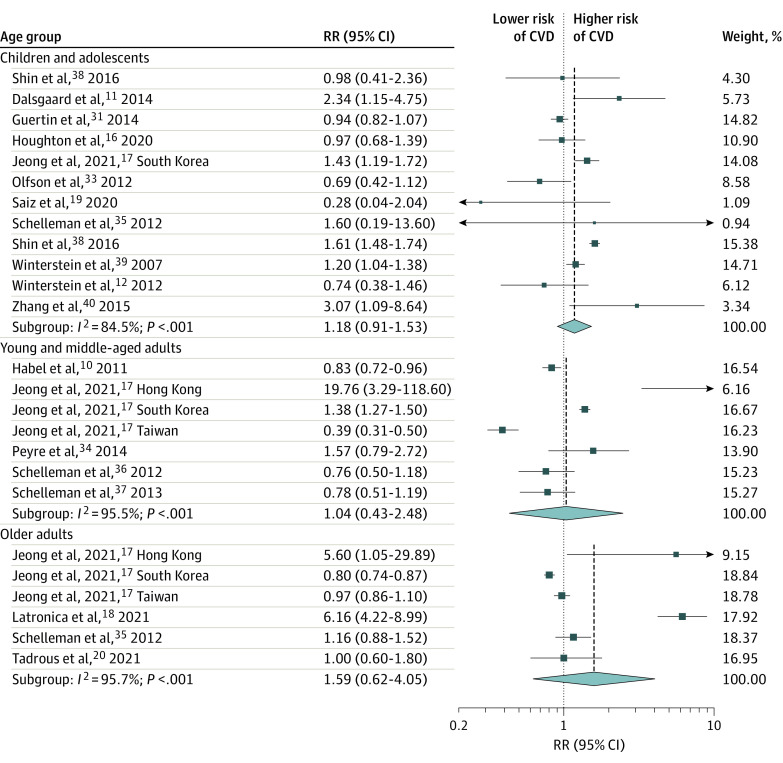
Risk of Any Cardiovascular Event by Age Group Among Individuals Receiving Attention-Deficit/Hyperactivity Disorder Medication CVD indicates cardiovascular disease; RR, risk ratio.

**Figure 3.**
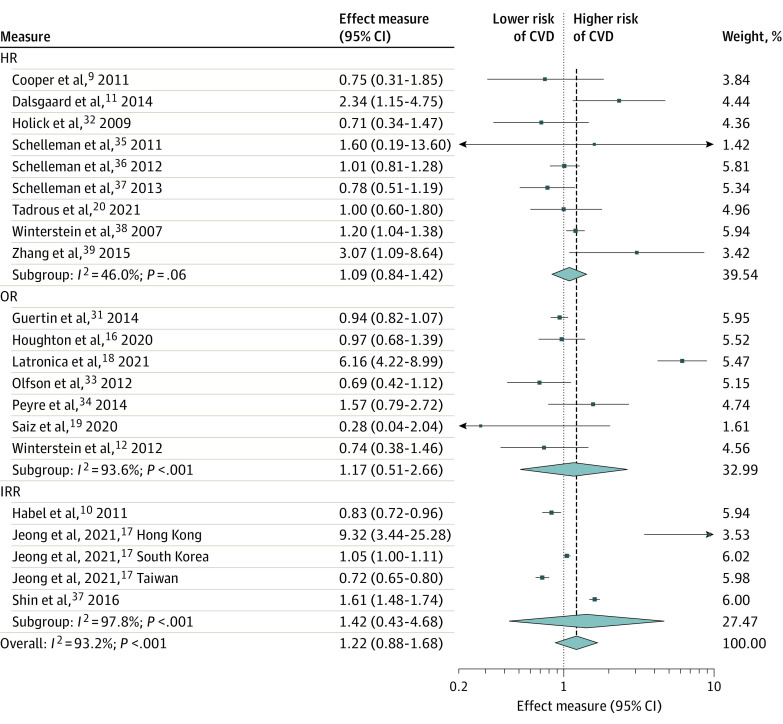
Risk of Any Cardiovascular Event by Measure of Association Among Individuals Receiving Attention-Deficit/Hyperactivity Disorder Medication CVD indicates cardiovascular disease; HR, hazard ratio; IRR, incidence rate ratio; OR, odds ratio.

We found that ADHD medication use was not statistically significantly associated with the risk of any CVD among children and adolescents (RR, 1.18; 95% CI, 0.91-1.53), young and middle-aged adults (RR, 1.04; 95% CI, 0.43-2.48), older adults (RR, 1.59; 95% CI, 0.62-4.05) (Figure 2), or overall (RR, 1.22; 95% CI, 0.88-1.68 (Figure 3). Analysis by effect measures also did not show significant estimates for HR (1.09; 95% CI, 0.84-1.42), OR (1.17; 95% CI, 0.51-2.66), or IRR (1.42; 95% CI, 0.43-4.68 (Figure 3). Heterogeneity between studies was high and significant (Cochran *Q* = 292.7; *P* < .001; *I*^2^ = 93.2%). When restricting to specific effect measurements, heterogeneity was not significant for the analysis with HR as effect measures, yet it was still significant in other subgroups (Figure 3). As shown in the leave-one-out sensitivity analysis (eFigure 1 in the Supplement), the estimate was not driven by a single study. There was no evidence of publication bias, and a small study effect for the primary outcomes (eFigures 2 and 3 in the Supplement).

In subgroup analyses, we found no statistically significant associations of stimulant (RR, 1.24; 95% CI, 0.84-1.83) and nonstimulant medications (RR, 1.22; 95% CI, 0.25-5.97) with any CVD (Table 2; eFigure 4 in the Supplement). When examining specific CVD outcomes (Table 2; eFigure 5 in the Supplement), no statistically significant associations were suggested for cardiac arrest or arrhythmias (RR, 1.60; 95% CI, 0.94-2.72), cerebrovascular diseases (RR, 0.91; 95% CI, 0.72-1.15), or myocardial infarction (RR, 1.06; 95% CI, 0.68-1.65). When examining stimulant medications, we found a similar pattern of results (eFigure 6 in the Supplement).

**Table 2.** Summary of Subgroup Analysis Results

Subgroup	Studies, No. (data sets, No.)	Pooled RRs	*P* ^a^	*I*^2^, %
Type of ADHD medication				
Stimulants	15 (17)	1.24 (0.84-1.83)	<.001	94.2
Nonstimulants	3 (3)	1.22 (0.25-5.97)	<.001	90.2
Specific CVD outcome				
Cardiac arrest or tachyarrhythmias	9 (9)	1.60 (0.94-2.72)	<.001	77.9
Cerebrovascular diseases	10 (10)	0.91 (0.72-1.15)	.14	34.0
Myocardial infarction	8 (10)	1.06 (0.68-1.65)	<.001	86.2
Sex groups				
Male	3 (5)	1.08 (0.32-3.67)	<.001	96.1
Female	3 (5)	1.88 (0.43-8.24)	<.001	85.6
Prior CVD history				
Without CVD history	10 (11)	0.99 (0.73-1.33)	<.001	98.7
With CVD history	7 (8)	1.31 (0.80-2.16)	<.001	99.0

Abbreviations: ADHD, attention-deficit/hyperactivity disorder; CVD, cardiovascular disease; RR, risk ratio.

^a^ *P* value associated to the Cochran *Q* statistic of heterogeneity.

There was no association between ADHD medication use and any CVD for female (RR, 1.88; 95% CI, 0.43-8.24) and male (RR, 1.08; 95% CI, 0.32-3.67) patients. (Table 2; eFigure 7 in the Supplement). We found no statistically significant associations in either individuals without a history of CVD (RR, 0.99; 95% CI, 0.73-1.33) or individuals with a history of CVD (RR, 1.31; 95% CI, 0.80-2.16) (Table 2). In particular, the only 2 studies^11,40^ with long-term follow-up both showed elevated risk (RR, 2.01; 95% CI, 1.98-2.06 and RR, 3.07; 95% CI, 1.09-8.64) in those with a history of CVD (eFigure 8 in the Supplement).

## Discussion

### Main Findings

To our knowledge, this is the most comprehensive systematic review and meta-analysis of longitudinal observational studies on the association between ADHD medication use and the risk of CVD. By pooling results of 19 studies, we found no statistically significant association between ADHD medication use and CVD among children and adolescents, young and middle-aged adults, or older adults, although the pooled RR did not exclude a modest risk increase, especially for the risk of cardiac arrest or tachyarrhythmias. We did not detect any difference in the cardiovascular risk between stimulant and nonstimulant ADHD medication use. There was no association between ADHD medication and any CVD among female patients and those with preexisting CVD, although further study may be needed in these populations.

This updated meta-analysis enabled us to include 13 more studies than the previous meta-analyses.^14,15^ Unlike the previous meta-analyses, we did not include all-cause mortality in our primary outcome (but sudden cardiac death), as previous studies have shown that most of the mortality in patients with ADHD was due to unnatural causes (eg, accidents and suicide).^41,42^ We examined a broad range of cardiovascular outcomes including important cardiovascular outcomes (eg, hypertension and heart failure) in addition to those examined in previous meta-analyses (cardiac arrest, tachyarrhythmias, myocardial infarction, and stroke). We found no statistically significant association between ADHD medication use and CVD among children and adolescents, young and middle-aged adults, or older adults, although the confidence interval could not exclude an increased risk. It should be noted that as the absolute risk is relatively low, even a significant RR of 22% risk increase in general would possibly be offset by the benefits of medications, eg, alleviating ADHD symptoms and reducing risky behavior.^6,43^ The trade-off between benefits and risks could be different in high-risk patients. Regarding specific cardiovascular outcomes, results from previous meta-analyses for specific cardiovascular outcomes (ie, cardiac arrest or tachyarrhythmias and stroke) are inconsistent.^14,15^ We found that ADHD medication use seemed to be associated with an increased risk of cardiac arrest or tachyarrhythmias, but not with cerebrovascular disease and myocardial infarction.

We also reported several findings that were not explored in previous meta-analyses. In terms of types of ADHD medication, we found both stimulant and nonstimulant ADHD medications were not statistically significantly associated with any CVD, with similar pooled RRs. These would suggest similar null effects on CVD or similar degree of confounding in studies of both stimulants and nonstimulants. We were unable to compare stimulants vs nonstimulants for the risk of specific CVD due to the limited number of studies that examined nonstimulants. Of note, 1 previous open-label extension of an RCT study^44^ compared the cardiovascular risks of a stimulant ADHD medication (dexmethylphenidate) vs a nonstimulant ADHD medication guanfacine. The study found that dexmethylphenidate was associated with increased systolic blood pressure, while guanfacine was associated with decreased heart rate, but both returned to baseline value during the 1-year open-label extension phase. It suggests that there might be differences in cardiovascular risks between stimulants and nonstimulants, but these differences may attenuate over time, thus not leading to a significant difference in clinically relevant outcomes. Nevertheless, head-to-head comparison studies based on observational data are warranted to compare stimulant vs nonstimulant ADHD medications regarding the risk of specific CVD.

We found that the risk of cardiovascular events associated with ADHD medications seemed to be higher among those with preexisting CVD compared with no prior CVD, although the findings did not reach the threshold for statistical significance. This coincides with raising concerns that individuals with congenital or acquired CVD are predisposed to additional risk.^45^ Despite the lack of data supporting CVD history as a contraindication for ADHD medications, the FDA labeling includes a warning on the use of ADHD medications among individuals with structural cardiac abnormalities or other serious heart problems. Current treatment guidelines generally recommend carefully assessing patients with ADHD (eg, personal and family history of CVD, physical examination, electrocardiogram) and identifying individuals at risk before initiating ADHD medications.^45^ Careful monitoring should also be performed after initiation.^46,47^ Further studies focusing on the potential modifying risk of preexisting CVD, ideally separating risks for congenital or acquired CVD, are warranted. Clinical guidelines on prescribing ADHD medications among high-risk individuals should be updated once further evidence is available. We also found the point estimates for risk of CVD seemed to be higher among female compared with male patients, although only 3 studies have examined the sex-specific association along with high heterogeneity between studies. Previous research has shown that females with ADHD have somewhat different patterns of comorbidities^48,49^ and response to stimulants^50^ than males, and additional research is needed to examine this potential sex difference.

### Methodology Consideration

The analysis of observational data provides an emerging opportunity to generate evidence to inform clinical decisions, but there are important issues to consider to avoid biases.^51^ One key issue is that treatment is not randomly assigned, which could result in confounded estimates. The included studies mainly reflected practice in clinical settings rather than controlled settings, so the prescription of ADHD medications is influenced by the clinician’s perception of CVD risk. Most studies adjusted for a range of measured confounders, but the included confounders varied across studies. Many studies adjusted for demographic characteristics, and several adjusted for baseline comorbid conditions (eg, psychosis, obesity, and diabetes) and comedications (eg, antiepileptics, antidepressants, and asthma medications), yet few studies accounted for time-varying confounding factors. Moreover, several studies used general population control (rather than individuals with ADHD) as the comparison group, but only 1 study^9^ adjusted for ADHD status. Not accounting for ADHD status would lead to bias, as recent research found that ADHD itself is a risk factor for CVD independent from comorbid psychiatric and somatic conditions.^52^

In addition, other fundamental flaws, such as selection bias and immortal time bias, need to be considered carefully when interpreting results from observational studies.^51^ Nine of the 19 included studies^9,10,11,12,16,18,19,34,40^ used prevalent users instead of incident users, and 7 studies^16,18,19,34,35,36,37^ were at risk of immortal time bias. Misclassification and exclusion of the so-called immortality period would necessarily bias the results toward favoring the treatment.^53^ Unlike lack of randomization, these flaws can be easily prevented by study design, eg, explicitly emulating a pragmatic target trial.^54^ Moreover, most of the included studies (17 of 19) had an average follow-up time of up to 2 years. Only 2 studies had sufficient follow-up time to examine the long-term cardiovascular risk associated with ADHD medication, but these studies were only moderate in their quality (GRACE score, 6 and 8 of 11). Thus, further studies with rigorous methods are needed to evaluate the long-term risk of CVD associated with ADHD medication use.

### Clinical Implications

Overall, our meta-analysis provides reassuring data on the putative cardiovascular risk with ADHD medications, but the possible associations with cardiac arrest or tachyarrhythmias, among female patients, and among those with preexisting CVD warrants further investigation. Importantly, our findings are presented at the population level; in clinical practice, specific individuals with ADHD might be particularly prone to negative cardiovascular outcomes; therefore, clinicians should discuss with their patients and families the possible cardiovascular risk of ADHD medication in light of the latest evidence, and they should rigorously follow clinical guidelines that suggest monitoring of blood pressure and heart rate at baseline and each medication review.

### Limitations

There are several limitations to consider when interpreting the results. First, heterogeneity was high and significant for most analyses. Although this heterogeneity does not invalidate our results, it indicates that the pooled RR cannot appropriately summarize results from all individual studies and should therefore be interpreted with caution.^55^ When restricting to specific cardiovascular outcomes, heterogeneity was not significant for the analysis on CVDs, yet it was still significant in the subgroup analyses by sex and preexisting CVD. Second, due to a lack of data, we were unable to compare the associations with specific ADHD medications. Third, as few studies have information on dosage and duration of medication use, investigation of the dose-response association was not possible. Fourth, although the GRACE checklist is validated for evaluating the quality of observational studies of medical treatment, a total score approach for risk of bias assessment needed to be validated. Additionally, most of the included studies were conducted in the United States and Europe, which means the results may not generalize to other settings.

## Conclusions

The results of this meta-analysis suggested no statistically significant association between ADHD medication use and the risk of any cardiovascular events across age groups, although a modest risk increase could not be excluded, especially for the risk of cardiac arrest or tachyarrhythmias. Our study also warrants future studies with rigorous study designs to investigate the risk of cardiovascular events among female patients and among those with preexisting CVD, as well as the long-term risk of ADHD medication use.

## Notes

Supplement. **eTable 1.** Search Strategy and Results From Each Electronic Database **eTable 2.** Items of the GRACE Checklist **eTable 3.** Studies Excluded From the Systematic Review After Full-Text Screen, With Reasons **eTable 4.** Absolute Risk and Risk Difference in CVD Outcomes Among the ADHD Medication Use Group vs the Reference Group **eTable 5.** Quality Assessment by GRACE Checklist **eFigure 1.** Results of Leave-One-Out Sensitivity Analysis **eFigure 2.** Results From Egger Test **eFigure 3.** Publication Bias of Included Studies **eFigure 4. **Associations of Stimulant and Nonstimulant ADHD Medication Use With CVD **eFigure 5. **Associations Between ADHD Medication Use and Specific CVD Outcomes **eFigure 6.** Associations Between Stimulant ADHD Medication Use and Specific CVD Outcomes **eFigure 7.** Associations Between ADHD Medication Use and CVD, by Sex Group **eFigure 8.** Associations Between ADHD Medication Use and CVD, by History of CVD
eReferences.
