# Supplementary material for: Risk of Cardiovascular Diseases Associated With Medications Used in Attention-Deficit/Hyperactivity Disorder: A Systematic Review and Meta-analysis
Source: JAMA Netw Open. 2022 Nov 23;5(11):e2243597. doi: 10.1001/jamanetworkopen.2022.43597 (PMC9685490; doi:10.1001/jamanetworkopen.2022.43597)
Supplement: Supplement. — eTable 1. Search Strategy and Results From Each Electronic Database eTable 2. Items of the GRACE Checklist eTable 3. Studies Excluded From the Systematic Review After Full-Text Screen, With Reasons eTable 4. Absolute Risk and Risk Difference in CVD Outcomes Among the ADHD Medication Use Group vs the Reference Group eTable 5. Quality Assessment by GRACE Checklist eFigure 1. Results of Leave-One-Out Sensitivity Analysis eFigure 2. Results From Egger Test eFigure 3. Publication Bias of Included Studies eFigure 4. Associations of Stimulant and Nonstimulant ADHD Medication Use With CVD eFigure 5. Associations Between ADHD Medication Use and Specific CVD Outcomes eFigure 6. Associations Between Stimulant ADHD Medication Use and Specific CVD Outcomes eFigure 7. Associations Between ADHD Medication Use and CVD, by Sex Group eFigure 8. Associations Between ADHD Medication Use and CVD, by History of CVD eReferences. [file jamanetwopen-e2243597-s001.pdf]

Supplemental Online Content

Zhang L, Yao H, Li L, et al. Risk of cardiovascular diseases associated with medications used in attention-deficit/hyperactivity disorder: a systematic review and meta-analysis. *JAMA Netw Open.* 2022;5(11):e2243597. doi:10.1001/jamanetworkopen.2022.43597

eTable 1. Search Strategy and Results From Each Electronic Database

eTable 2. Items of the GRACE Checklist

eTable 3. Studies Excluded From the Systematic Review After Full-Text Screen, With Reasons

eTable 4. Absolute Risk and Risk Difference in CVD Outcomes Among the ADHD Medication Use Group vs the Reference Group

eTable 5. Quality Assessment by GRACE Checklist

eFigure 1. Results of Leave-One-Out Sensitivity Analysis

eFigure 2. Results From Egger Test

eFigure 3. Publication Bias of Included Studies

eFigure 4. Associations of Stimulant and Nonstimulant ADHD Medication Use With CVD

eFigure 5. Associations Between ADHD Medication Use and Specific CVD Outcomes

eFigure 6. Associations Between Stimulant ADHD Medication Use and Specific CVD Outcomes

eFigure 7. Associations Between ADHD Medication Use and CVD, by Sex Group

eFigure 8. Associations Between ADHD Medication Use and CVD, by History of CVD

eReferences.

This supplemental material has been provided by the authors to give readers additional information about their work.

© 2022 Zhang L et al. *JAMA Network Open.*

eTable 1. Search Strategy and Results From Each Electronic Database

Electronic database	Search terms	Results
PubMed	("cardiovascular diseases"[MeSH Terms] OR "cardiovascular system"[MeSH Terms] OR "coronary disease"[MeSH Terms] OR "heart diseases"[MeSH Terms] OR "death, sudden"[MeSH Terms] OR "death, sudden, cardiac"[MeSH Terms] OR "arrhythmias, cardiac"[MeSH Terms] OR "tachycardia"[MeSH Terms] OR "myocardial infarction"[MeSH Terms] OR "hypertension"[MeSH Terms] OR ("myocardial ischemia"[MeSH Terms] OR "coronary artery disease"[MeSH Terms]) OR "coronary artery disease"[MeSH Terms] OR "heart failure"[MeSH Terms] OR "heart arrest"[MeSH Terms] OR "myocarditis"[MeSH Terms] OR "angina pectoris"[MeSH Terms] OR "cardiomyopathies"[MeSH Terms] OR "peripheral arterial disease"[MeSH Terms] OR "ischemic attack, transient"[MeSH Terms] OR "cerebrovascular disorders"[MeSH Terms] OR (("cerebrum"[MeSH Terms] OR "brain"[MeSH Terms]) AND "vascular diseases"[MeSH Terms]) OR "stroke"[MeSH Terms] OR "cardiovascular disease"[Title/Abstract] OR "cardiovascular diseases"[Title/Abstract] OR "cardiovascular event"[Title/Abstract] OR "cardiovascular events"[Title/Abstract] OR "cardiovascular disorder"[Title/Abstract] OR "cardiovascular disorders"[Title/Abstract] OR "coronary heart disease"[Title/Abstract] OR "coronary heart diseases"[Title/Abstract] OR "heart diseases"[Title/Abstract] OR "heart disease"[Title/Abstract] OR "sudden death"[Title/Abstract] OR "sudden cardiac death"[Title/Abstract] OR "arrhythmia"[Title/Abstract] OR "tachycardia"[Title/Abstract] OR "tachyarrhythmia"[Title/Abstract] OR "myocardial infarction"[Title/Abstract] OR "heart attack"[Title/Abstract] OR "hypertension"[Title/Abstract] OR "hypertensive"[Title/Abstract] OR "ischemic heart disease"[Title/Abstract] OR "heart failure"[Title/Abstract] OR "cardiac arrest"[Title/Abstract] OR "myocarditis"[Title/Abstract] OR "angina"[Title/Abstract] OR "cardiomyopathy"[Title/Abstract] OR "peripheral artery disease"[Title/Abstract] OR "peripheral artery diseases"[Title/Abstract] OR "transient ischemic attack"[Title/Abstract] OR "transient ischemic attacks"[Title/Abstract] OR "transient ischaemic attack"[Title/Abstract] OR "transient ischaemic attacks"[Title/Abstract] OR "cerebrovascular disease"[Title/Abstract] OR "cerebrovascular diseases"[Title/Abstract] OR "cerebro vascular disease"[Title/Abstract] OR "cerebro vascular diseases"[Title/Abstract] OR "cerebral vascular disease"[Title/Abstract] OR "cerebral vascular diseases"[Title/Abstract] OR "stroke"[Title/Abstract]) AND ("attention deficit disorder with hyperactivity"[MeSH Terms] OR "central nervous system stimulants"[MeSH Terms] OR "methylphenidate"[MeSH Terms] OR "dexmethylphenidate hydrochloride"[MeSH Terms] OR "methamphetamine"[MeSH Terms] OR "dextroamphetamine"[MeSH Terms] OR "amphetamine"[MeSH Terms] OR "amphetamines"[MeSH Terms] OR "lisdexamfetamine dimesylate"[MeSH Terms] OR "atomoxetine hydrochloride"[MeSH Terms] OR "guanfacine"[MeSH Terms] OR "clonidine"[MeSH Terms] OR "viloxazine"[MeSH Terms] OR "ADHD"[Title/Abstract] OR "attention deficit hyperactivity disorder"[Title/Abstract] OR "attention-deficit"[Title/Abstract] OR "hyperkinetic disorder"[Title/Abstract] OR "hyperkinetic syndrome"[Title/Abstract] OR "psychostimulant"[Title/Abstract] OR "psychostimulants"[Title/Abstract] OR "central nervous system stimulant"[Title/Abstract] OR "central nervous system stimulants"[Title/Abstract] OR "stimulant"[Title/Abstract] OR "stimulants"[Title/Abstract] OR "non-stimulant"[Title/Abstract] OR "non-stimulants"[Title/Abstract] OR "methylphenidate"[Title/Abstract] OR "dexmethylphenidate"[Title/Abstract] OR "methamphetamine"[Title/Abstract] OR "dextroamphetamine"[Title/Abstract] OR "amphetamine"[Title/Abstract] OR "amphetamines"[Title/Abstract] OR "lisdexamfetamine"[Title/Abstract] OR "atomoxetine"[Title/Abstract] OR "guanfacine"[Title/Abstract] OR "clonidine"[Title/Abstract] OR "viloxazine"[Title/Abstract]) AND ("epidemiology"[MeSH Terms] OR "cohort studies"[MeSH Terms] OR "case control studies"[MeSH Terms] OR "longitudinal studies"[MeSH Terms] OR "retrospective studies"[MeSH Terms] OR "population"[MeSH Terms] OR "population groups"[MeSH Terms] OR "registries"[MeSH Terms] OR "records"[MeSH Terms] OR "epidemiolog*"[Title/Abstract] OR "observational"[Title/Abstract] OR "cohort"[Title/Abstract] OR	2063 hits

© 2022 Zhang L et al. *JAMA Network Open.*

	"case-control"[Title/Abstract] OR "case-control"[Title/Abstract] OR "follow-up"[Title/Abstract] OR "follow-up"[Title/Abstract] OR "longitudinal"[Title/Abstract] OR "prospective"[Title/Abstract] OR "retrospective"[Title/Abstract] OR "population*"[Title/Abstract] OR "regist*"[Title/Abstract] OR "claims"[Title/Abstract] OR "record"[Title/Abstract])	
Embase and Medline	#1 'cardiovascular disease':ab,ti OR 'cardiovascular diseases':ab,ti OR 'cardiovascular event':ab,ti OR 'cardiovascular events':ab,ti OR 'cardiovascular disorder':ab,ti OR 'cardiovascular disorders':ab,ti OR 'coronary heart disease':ab,ti OR 'coronary heart diseases':ab,ti OR 'heart diseases':ab,ti OR 'heart disease':ab,ti OR 'sudden death':ab,ti OR 'sudden cardiac death':ab,ti OR arrhythmia:ab,ti OR tachycardia:ab,ti OR tachyarrhythmia:ab,ti OR 'myocardial infarction':ab,ti OR 'heart attack':ab,ti OR hypertension:ab,ti OR hypertensive:ab,ti OR 'ischemic heart disease':ab,ti OR 'heart failure':ab,ti OR 'cardiac arrest':ab,ti OR myocarditis:ab,ti OR angina:ab,ti OR cardiomyopathy:ab,ti OR 'peripheral artery disease':ab,ti OR 'peripheral artery diseases':ab,ti OR 'transient ischemic attack':ab,ti OR 'transient ischemic attacks':ab,ti OR 'transient ischaemic attack':ab,ti OR 'transient ischaemic attacks':ab,ti OR 'cerebrovascular disease':ab,ti OR 'cerebrovascular diseases':ab,ti OR 'cerebro vascular disease':ab,ti OR 'cerebro vascular diseases':ab,ti OR 'cerebral vascular disease':ab,ti OR 'cerebral vascular diseases':ab,ti OR stroke:ab,ti<br><br>#2 adhd:ab,ti OR 'attention-deficit hyperactivity disorder':ab,ti OR 'attention deficit':ab,ti OR 'hyperkinetic disorder':ab,ti OR 'hyperkinetic syndrome':ab,ti OR psychostimulant:ab,ti OR psychostimulants:ab,ti OR 'central nervous system stimulant':ab,ti OR 'central nervous system stimulants':ab,ti OR stimulant:ab,ti OR stimulants:ab,ti OR 'non stimulant':ab,ti OR 'non stimulants':ab,ti OR methylphenidate:ab,ti OR dexmethylphenidate:ab,ti OR methamphetamine:ab,ti OR dextroamphetamine:ab,ti OR amphetamine:ab,ti OR amphetamines:ab,ti OR lisdexamfetamine:ab,ti OR atomoxetine:ab,ti OR guanfacine:ab,ti OR clonidine:ab,ti OR viloxazine:ab,ti<br><br>#3 epidemiolog*:ab,ti OR observational:ab,ti OR cohort:ab,ti OR 'case control':ab,ti OR 'case-control':ab,ti OR 'follow up':ab,ti OR 'follow-up':ab,ti OR longitudinal:ab,ti OR prospective:ab,ti OR retrospective:ab,ti OR population*:ab,ti OR regist*:ab,ti OR claims:ab,ti OR record:ab,ti<br><br>#1 AND #2 AND #3	1953 hits
PscyINFO	1. (cardiovascular disease or cardiovascular diseases or cardiovascular event or cardiovascular events or cardiovascular disorder or cardiovascular disorders or coronary heart disease or coronary heart diseases or heart diseases or heart disease or sudden death or sudden cardiac death or arrhythmia or tachycardia or tachyarrhythmia or myocardial infarction or heart attack or hypertension or hypertensive or ischemic heart disease or heart failure or cardiac arrest or myocarditis or angina or cardiomyopathy or peripheral artery disease or peripheral artery diseases or transient ischemic attack or transient ischemic attacks or transient ischaemic attack or transient ischaemic attacks or cerebrovascular disease or cerebrovascular diseases or cerebro vascular disease or cerebro vascular diseases or cerebral vascular disease or cerebral vascular diseases or stroke).ab,ti.<br><br>2. (ADHD or attention-deficit hyperactivity disorder or attention deficit or hyperkinetic disorder or hyperkinetic syndrome or psychostimulant or psychostimulants or central nervous system stimulant or central nervous system stimulants or stimulant or stimulants or non-stimulant or non-stimulants or methylphenidate or dexmethylphenidate or methamphetamine or dextroamphetamine or amphetamine or amphetamines or lisdexamfetamine or atomoxetine or guanfacine or clonidine or viloxazine).ab,ti.<br><br>3. (epidemiolog* or observational or cohort or case control or case-control or follow up or follow-up or longitudinal or prospective or retrospective or population* or regist* or claims or record).ab,ti.<br><br>4. 1 and 2 and 3	1113 hits

© 2022 Zhang L et al. *JAMA Network Open.*

Web of Science	TS=(cardiovascular disease OR cardiovascular diseases OR cardiovascular event OR cardiovascular events OR cardiovascular disorder OR cardiovascular disorders OR coronary heart disease OR coronary heart diseases OR heart diseases OR heart disease OR sudden death OR sudden cardiac death OR arrhythmia OR tachycardia OR tachyarrhythmia OR myocardial infarction OR heart attack OR hypertension OR hypertensive OR ischemic heart disease OR heart failure OR cardiac arrest OR myocarditis OR angina OR cardiomyopathy OR peripheral artery disease OR peripheral artery diseases OR transient ischemic attack OR transient ischemic attacks OR transient ischaemic attack OR transient ischaemic attacks OR cerebrovascular disease OR cerebrovascular diseases OR cerebro vascular disease OR cerebro vascular diseases OR cerebral vascular disease OR cerebral vascular diseases OR stroke) AND TS=(ADHD OR attention-deficit hyperactivity disorder OR attention deficit OR hyperkinetic disorder OR hyperkinetic syndrome OR psychostimulant OR psychostimulants OR central nervous system stimulant OR central nervous system stimulants OR stimulant OR stimulants OR non-stimulant OR non-stimulants OR methylphenidate OR dexmethylphenidate OR methamphetamine OR dextroamphetamine OR amphetamine OR amphetamines OR lisdexamfetamine OR atomoxetine OR guanfacine OR clonidine OR viloxazine) AND TS=(epidemiolog* OR observational OR cohort OR case control OR case-control OR follow up OR follow-up OR longitudinal OR prospective OR retrospective OR population* OR regist* OR claims OR record)	2664 hits

© 2022 Zhang L et al. *JAMA Network Open.*

eTable 2. Items of the GRACE Checklist

**Q. The Good Research for Comparative Effectiveness (GRACE) Checklist v5.0 (last amended in 2016)**<br>**Website:** https://www.graceprinciples.org/		
Major Components	Response options	
Data		
D1. Were treatment and/or important details of treatment exposure adequately recorded for the study purpose in the data source(s)?	+	-
D2. Were the primary outcomes adequately recorded for the study purpose (e.g., available in sufficient detail through data source(s))?	+	-
D3. Was the primary clinical outcome(s) measured objectively rather than subject to clinical judgment (e.g., opinion about whether the patient's condition has improved)?	+	-
D4. Were primary outcomes validated, adjudicated, or otherwise known to be valid in a similar population?	+	-
D5. Was the primary outcome(s) measured or identified in an equivalent manner between the treatment/ intervention group and the comparison group(s)?	+	-
D6. Were important covariates that may be known confounders or effect modifiers available and recorded?	+	-
Methods		
M1. Was the study (or analysis) population restricted to new initiators of treatment or those starting a new course of treatment?	+	-
M2. If one or more comparison groups were used, were they concurrent comparators? If not, did the authors justify the use of historical comparisons group(s)?	+	-
M3. Were important covariates, confounding and effect modifying variables taken into account in the design and/or analysis?	+	-
M4. Is the classification of exposed and unexposed person-time free of "immortal time bias"?	+	-
M5. Were any meaningful analyses conducted to test key assumptions on which primary results are based?	+	-

© 2022 Zhang L et al. *JAMA Network Open.*

eTable 3. Studies Excluded From the Systematic Review After Full-Text Screen, With Reasons

Reference	Reason(s) for exclusion
Acquarone-Greiwe, D., et al. (2009). Methylphenidate: Harmless intoxication but severe side effects? *Clinical Toxicology,* 47**,** 457.	No comparison group
Alrwisan, A. A., et al. (2019). Concomitant use of quinolones and stimulants and the risk of cardiovascular adverse events: A comparative safety study. *Pharmacoepidemiology and Drug Safety,* 28**,** 405.	Not related to our topic. It is mainly focused on the association between stimulant-antibiotic combinations and CVD
Alrwisan, A. A., et al. (2019b). Concomitant Use of Quinolones and Stimulants and the Risk of Adverse Cardiovascular Symptoms: A Retrospective Cohort Study. *Pharmacotherapy,* 39**,** 1167-1178.	Not related to our topic. It mainly focuses on the association between stimulant-antibiotic combinations and CVD
Bali, V., et al. (2019). Cardiovascular Safety of Concomitant Use of Atypical Antipsychotics and Long-Acting Stimulants in Children and Adolescents With ADHD. *J Atten Discord,* 23**,** 163-172.	Not related to our topic. It mainly focuses on the association between long-acting stimulant-atypical antipsychotics combinations and CVD
Childress, A. C. (2019). Safety of Extended-Release Methylphenidate in Preschool Children With ADHD. Journal of the American Academy of Child and Adolescent Psychiatry, 58(10), S296. doi:10.1016/j.jaac.2019.09.006	The outcome does not meet the inclusion criteria
Childress, A. C., et al. (2021). Long-Term Treatment With Extended-Release Methylphenidate Treatment in Children Aged 4 to <6 Years. *J Am Acad Child Adolesc Psychiatry*.	Clinical trial; No comparison group
Chin, K. M., et al. (2006). Is methamphetamine use associated with idiopathic pulmonary arterial hypertension? *Chest,* 130**,** 1657-1663.	No comparison group; Not related to our topic. It is a case series study, only describing the characteristic of PAH patients with stimulants using a history
Cilsal, E., et al. (2020). Early Cardiovascular Evaluation after Methylphenidate in Children with Attention-Deficit Hyperactivity Disorder. *Gazi Medical Journal,* 31**,** 345-348.	Within patients comparison, but no effect size; Only comparing the clinical indicator before and after using ADHD medication, no detailed description about abnormal clinical indicator
Conzelmann, A., et al. (2019). Long-term cardiovascular safety of psychostimulants in children with attention deficit hyperactivity disorder. *International Journal of Psychiatry in Clinical Practice,* 23**,** 157-159.	Could not retrieve the effect measure, because this study only reported continuous measures of heart rate, blood pressure
Cortese, S., et al. (2015). Safety of Methylphenidate and Atomoxetine in Children with Attention-Deficit/Hyperactivity Disorder (ADHD): Data from the Italian National ADHD Registry. *CNS Drugs,* 29**,** 865-877.	No comparison group
Curran, L. A., et al. (2019). Clinical correlates and outcomes of methamphetamine-associated cardiovascular disease among hospitalized patients in California. *European Heart Journal,* 40**,** 1350.	Abstract only and it is about drug abuse
Dalsgaard, S., et al. (2011). Long-term cardiac adverse effects of ADHD medication in children and adolescents: A nationwide register-based follow-up study. *European Child and Adolescent Psychiatry,* 20**,** S107.	Duplicate (Conference)
Dalsgaard, S., et al. (2015). Cardiovascular safety of psychostimulants in children with ADHD: Findings from a population-based cohort study. *ADHD Attention Deficit and Hyperactivity Disorders,* 7**,** S14.	Duplicate (conference abstract)
Darke, S., et al. (2019). Psychostimulant Use and Fatal Stroke in Young Adults. *J Forensic Sci,* 64**,** 1421-1426.	No comparison group; Include both illicit and licit use in this study

© 2022 Zhang L et al. *JAMA Network Open.*

Darracq, M. A., et al. (2021). Sustained stimulation? Characteristics of modified release and immediate release stimulant exposures reported to the national poison data system. *Clin Toxicol (Phila)*, 59, 200-207.	Not observational study
Davies, M., et al. (2011). Risk of cardiac events in patients taking atomoxetine: Results of a matched cohort analysis. *Drug Safety,* 34**,** 981.	Insufficient data available
Davis, L. E., et al. (2017). Diagnoses of cardiovascular disease or addiction in U.S. adults treated for ADHD with stimulants or atomoxetine: Is use consistent with product labelling? *Pharmacotherapy,* 37**,** e142.	Duplicate (Conference)
Dovies, M., et al. (2009). A study to examine cardiac events in patients prescribed atomoxetine in England: Results of an interim modified prescription event monitoring study. Drug Safety, 32(10), 976-977.	No comparison group
Fairman, K. A., et al. (2018). Diagnoses of Cardiovascular Disease or Substance Addiction/Abuse in US Adults Treated for ADHD with Stimulants or Atomoxetine: Is Use Consistent with Product Labelling? *Drugs Real World Outcomes,* 5**,** 69-79.	Focused on contraindication, CVD prevalence accessed 12 months before ADHD medication initiation; No comparison group, no effect size
Gomez-Lumbreras, A., et al. (2018). NERVOUS SYSTEM DRUGS AND RISK OF ISCHEMIC STROKE: A REAL WORLD DATA CASE-CONTROL STUDY. *Value in Health,* 21**,** S93.	Only part of the conference abstract was published, which focused on antiepileptics
Gould, M. S., et al. (2009). Sudden Death and Use of Stimulant Medications in Youths. *American Journal of Psychiatry,* 166**,** 992-1001.	Not focusing on CVD, include unknown reasons for sudden death in cases
Hadinezhad, P., et al. (2019). Study of Methamphetamine Use in Patients Referred to Emergency Ward of a General Hospital at North of Iran in 2017. *Addict Health,* 11**,** 18-25.	Exposure is not a pharmacological treatment of ADHD medications in this study, because whether an individual is exposed to drugs or not was defined by a urine test (positive or negative); No comparison group, no effect size
Hennessy, S., et al (2010). Cardiovascular safety of ADHD medications: rationale for and design of an investigator-initiated observational study. Pharmacoepidemiology and drug safety, 19(9), 934–941.	Duplicate; It is a protocol
Hills, N. K., et al. (2014). Stimulant medications as a risk factor for childhood stroke. Stroke, 45.	Insufficient data available
Houghton, R., et al. (2019). 1.28 ASSESSMENT OF ADHD MEDICATION USE AND ASSOCIATIONS WITH SERIOUS CARDIOVASCULAR EVENTS IN CHILDREN AND ADOLESCENTS WITH ASD IN THE UNITED STATES. *Journal of the American Academy of Child and Adolescent Psychiatry,* 58**,** S155-S156.	Duplicate (Conference)
Huang, M. C., et al. (2016). Risk of Cardiovascular Diseases and Stroke Events in Methamphetamine Users: A 10-Year Follow-Up Study. *J Clin Psychiatry,* 77**,** 1396-1403.	The study population (with a diagnosis of drug dependence) is not our target population; Drug abuse
Jain, S., et al. (2017). Association of stimulant use with IPAH: A case-control study. American Journal of Respiratory and Critical Care Medicine, 195. doi:10.1164/ajrccm-conference.2017.A18	Exposure does not meet the criteria
Jeong, H. E., et al. (2020). No association between methylphenidate use and myocardial infarction: A multinational self-controlled case series study. *Pharmacoepidemiology and Drug Safety,* 29**,** 580-580.	Duplicate (Conference)
Kuehn, B. M. (2009). Stimulant use is linked to sudden death in children without heart problems. *Jama,* 302**,** 613-614.	It is medical News & Perspectives

Larsson, P. G., et al. (2015). Incidence of bradycardia at arrival to the operating room after oral or intravenous premedication with clonidine in children. *Paediatr Anaesth,* 25**,** 956-962.	The incidence of bradycardia following oral or intravenous premedication with clonidine in a pediatric population scheduled for anesthesia is low.
Lejdstrom, R. B., et al. (2012). Trends in paediatric ADHD drug prescription in the UK and cardiovascular event rates. *Pharmacoepidemiology and Drug Safety,* 21**,** 136.	In the full text they no longer mentioned the CVD risks
Mau, M. K., et al. (2009). Risk factors associated with methamphetamine use and heart failure among native Hawaiians and other Pacific Island peoples. *Vasc Health Risk Manag,* 5**,** 45-52.	Not related topic: This study examined risk factors of methamphetamine use among heart failure patients; The exposure definition does not meet our criteria. positive MU use was defined in part by the toxicity test
Mccarthy, S., et al. (2009). Mortality associated with attention-deficit hyperactivity disorder (ADHD) drug treatment: a retrospective cohort study of children, adolescents and young adults using the general practice research database. *Drug Saf,* 32**,** 1089-1096.	Not focusing on CVD, No results on CVD-specific death
Mosholder, A. D., et al. (2016). Heart failure and cardiomyopathy following initiation of medications for attention-deficit/hyperactivity disorder. *Journal of the American Academy of Child and Adolescent Psychiatry,* 55**,** S168.	Duplicate (Conference)
Mosholder, A. D., et al. (2018). Incidence of heart failure and cardiomyopathy following initiation of medications for attention-deficit/hyperactivity disorder: a descriptive study. Journal of Clinical Psychopharmacology, 38, 505-508.	No non-use comparison group
Panei, P., et al. (2010). Safety of psychotropic drug prescribed for attention-deficit/hyperactivity disorder in Italy. *Adverse Drug Reaction Bulletin***,** 999-1002.	Lack of no-use group
Petitti, D. B., et al. (1998). Stroke and cocaine or amphetamine use. *Epidemiology,* 9**,** 596-600.	It is about drug abuse
Potey, C., et al. (2018). Cardiovascular safety of methylphenidate in adult attention deficit hyperactivity disorder (ADHD): The Lille experience. Fundamental and Clinical Pharmacology, 32, 24.	No comparison group
Prohaska, C. C., et al. (2021). Regional Variation in Methamphetamine-associated Pulmonary Arterial Hypertension: Who'd Better Call Saul? *Ann Am Thorac Soc,* 18**,** 584-585.	It is an editorial
Ramphul, K., et al. (2019). Cocaine, Amphetamine, and Cannabis Use Increases the Risk of Acute Myocardial Infarction in Teenagers. *Am J Cardiol,* 123**,** 354.	The exposure definition does not meet our criteria, it is a short report of drug abuse
Sayer, G. R., et al. (2016). Acute and long-term cardiovascular effects of stimulant, guanfacine, and combination therapy for attention-deficit/hyperactivity disorder. *Journal of Child and Adolescent Psychopharmacology,* 26**,** 882-888.	Duplicate, The study design does not meet our criteria, this is an RCT
Schelleman, H., et al. (2011a). ADHD medications and risk of serious cardiovascular events in adults. *Pharmacoepidemiology and Drug Safety,* 20**,** S122.	Duplicate (Conference)
Schelleman, H., et al. (2011b). Cardiovascular safety of ADHD medications in children and adolescents. *Pharmacoepidemiology and Drug Safety,* 20**,** S134.	Duplicate (Conference)
Shin, J. Y., et al. (2015). Cardiac risk associated with methylphenidate in paediatric patients with attention deficit hyperactivity disorder (ADHD): Self-controlled case series study in Korea. *Pharmacoepidemiology and Drug Safety,* 24**,** 235-236.	Duplicate (Conference)

© 2022 Zhang L et al. *JAMA Network Open.*

Stewart, E. M., et al. (2017). Risk of pulmonary hypertension in users of prescription amphetamine-based stimulants: A single center experience. American Journal of Respiratory and Critical Care Medicine, 195.	Exposure does not meet the criteria
Stewart, E. M., et al. (2017). Uncovering a link between prescription amphetamine-based stimulants and pulmonary hypertension-data from a northern California multi-specialty, mixed payer, health care system. American Journal of Respiratory and Critical Care Medicine, 195.	Duplicate (Conference)
Tadrous, M., et al. (2019). Use of stimulants in adults and risk of cardiovascular events: A multi-design approach. *Pharmacoepidemiology and Drug Safety,* 28**,** 19.	Duplicate (Protocol)
Tai, Y.-M., et al. (2012). Cardiovascular events and methylphenidate use in Taiwan. Journal of the American Academy of Child & Adolescent Psychiatry, 51(3), 324-325.	Insufficient data available
Trenque, T., et al. (2009). Spontaneous reporting with methylphenidate: Insufficient data. Drug Safety, 32(10), 983.	No comparison group
Weisler, R. H., et al. (2005). Long-term cardiovascular effects of mixed amphetamine salts extended release in adults with ADHD. *CNS Spectrums,* 10**,** 35-43.	Could not retrieve effect measure, because this study only reported continuous measure of heart rate, and blood pressure; No non-use comparison group
Wernicke, J. F., et al. (2003). Cardiovascular effects of atomoxetine in children, adolescents, and adults. Drug Safety, 26, 729-740.	Not observational study, cardiovascular effects described in RCT, not in extension
Winterstein, A. G., et al. (2009). Cardiac safety of methylphenidate versus amphetamine salts in the treatment of ADHD. *Pediatrics,* 124**,** e75-80.	No non-use comparison group
Yang, C. L., et al. (2010). Side effects associated with prescription of methylphenidate in Taiwan. *Value in Health,* 13**,** A103.	Insufficient data available
Yeo, K. K., et al. (2007). The association of methamphetamine use and cardiomyopathy in young patients. *Am J Med*, 120, 165-171	Unrelated topic
Vitiello B, et al (2012). Blood pressure and heart rate over 10 years in the multimodal treatment study of children with ADHD. Am J Psychiatry. 2012 Feb;169(2):167-77.	The outcome does not meet the inclusion criteria
García Ron, A., et al. (2021). Impacto del tratamiento con metilfenidato sobre las propiedades funcionales y estructurales del ventrículo izquierdo a medio plazo en el trastorno por déficit de atención e hiperactividad [The impact of methylphenidate treatment on the functional and structural properties of the left ventricle: A medium-term prospective study]. Anales de pediatria, S1695-4033(20)30537-3.	The outcome does not meet the inclusion criteria
Masi, G., et al. (2022). Acute Tolerability of Methylphenidate in Treatment-Naïve Children with ADHD: An Analysis of Naturalistically Collected Data from Clinical Practice. Pediatric drugs, 24(2), 147–154.	No comparison
Cohen, Audrey et al. (2022). Abstract TP206: Vascular Risk Factors And Stimulant Use Among Stroke Patients. Stroke. 53. 10.1161/str.53.suppl_1.TP206.	Exposure does not the meet criteria

© 2022 Zhang L et al. *JAMA Network Open.*

eTable 4. Absolute Risk and Risk Difference in CVD Outcomes Among the ADHD Medication Use Group vs the Reference Group

Study	Design	Measure	Number of participants	Number of cases	Exposed (ADHD medication) group				Unexposed (non-user) group				Risk difference	
					N	No. of event	Event rate[a]	Event %	N	No. of event	Event rate[a]	Event %	Event rate[a]	Event %
Habel et al,[1] 2011	Cohort	IRR	443198	1625	150359	234	218	0.16	292839	1391	261	0.48	-43	-0.32
Holick et al,[2] 2009	Cohort	HR	86205	65	43212	37	58.8	0.09	42993	28	42.5	0.06	16.3	0.03
Olfson et al,[3] 2012	Cohort	OR	171126	101	89031	21	0.92	0.06	82095	80	1.55	0.01	-0.63	0.05
Schelleman et al,[4] 2012	Cohort	HR	219954	1740	43999	98	3.40	0.22	175955	1642	3.55	0.93	-0.15	-0.71
Schelleman et al,[5] 2013	Cohort	HR	192905	572	38586	25	2.62	0.06	154319	547	3.03	0.35	-0.41	-0.29
Tadrous et al,[6] 2021	Cohort	HR	31310	932	6457	112	5.11	1.73	24853	820	3.66	3.30	1.45	-1.57
Winterstein et al,[7] 2012	Cohort	OR	1219847	95	386584	20	2.6	0.002	833263	75	5.0	0.02	-2.4	-0.02
Zhang et al,[8] 2015[b]	Cohort	HR	144	32	48	17	-	35.4	96	15	-	15.6	-	19.8
Latronica et al,[9] 2021	Cohort	OR	13233	236	4966	191	-	3.85	8267	45	-	0.54	-	3.31
Peyre et al,[10] 2014	Cohort	OR	807	76	216	27	-	12.5	591	49	-	8.29	-	4.21
Winterstein et al,[11] 2007	Cohort	HR	55383	830	32807	406	953	-	22576	424	909	1.88	44	-
Cooper et al,[12] 2011	Cohort	HR	1200438	56	-	7	1.87	-	-	49	3.07	-	-1.2	-
Dalsgaard et al,[13] 2014	Cohort	HR	8300	111	-	-	-	-	-	-	-	-	-	-
Schelleman et al,[14] 2011	Cohort	HR	241417	155	-	-	-	-	-	-	-	-	-	-
Guertin et al,[15] 2014	NCC[c]	OR	38495	1344	-	-	-	-	-	-	-	-	-	-
Houghton et al,[16] 2019	NCC	OR	2046	186	-	-	-	-	-	-	-	-	-	-
Saiz et al,[17] 2019	NCC	OR	2882	262	-	-	-	-	-	-	-	-	-	-
Shin et al,[18] 2016	SCCS[c]	IRR	1224	1224	-	-	-	-	-	-	-	-	-	-
Jeong et al,[19] 2021 South Korea	SCCS	IRR	2104	2104	-	-	-	-	-	-	-	-	-	-
Jeong et al,[19] 2021 Taiwan	SCCS	IRR	484	484	-	-	-	-	-	-	-	-	-	-
Jeong et al,[19] 2021 Hongkong	SCCS	IRR	30	30	-	-	-	-	-	-	-	-	-	-

Abbreviations: ADHD, attention-deficit/hyperactivity disorder; CVD, cardiovascular disease; NCC, Nested case-control; SCCS, Self-control case series

[a] Event rate present as the number of events per 100,000 person-years

[b] The study was conducted in a patient group with Long QT syndrome; thus the base rate of CVD event is high

[c] In nested case-control and self case series studies, data are not available to calculate the incidence rate of the disease being studied

© 2022 Zhang L et al. *JAMA Network Open.*

eTable 5. Quality Assessment by GRACE Checklist

Study	Data							Methods						Total
	D1	D2	D3	D4	D5	D6	Subtotal	M1	M2	M3	M4	M5	Subtotal	
Cooper (2011)	+	+	+	+	+	+	6	+	+	+	+	+	5	11
Dalsgaard (2014)	+	+	+	-	+	+	5	-	+	+	+	-	3	8
Guertin (2014)	+	+	+	-	+	-	4	+	+	-	+	+	4	8
Habel (2011)	+	+	+	+	+	+	6	+	+	+	+	+	5	11
Holick (2009)	+	+	+	-	+	+	5	+	+	+	+	-	4	9
Houghton (2019)	+	+	+	+	+	+	6	-	+	+	-	+	3	9
Jeong (2021)	+	+	+	+	+	+	6	+	+	+	+	-	4	10
Latronica (2021)	+	+	+	-	+	+	5	-	+	+	-	-	2	7
Olfson (2012)	+	+	+	+	+	-	5	+	+	+	+	-	4	9
Peyre (2014)	-	+	-	-	+	+	3	-	+	+	-	-	2	5
Saiz (2019)	+	+	+	+	+	+	6	-	+	+	-	+	3	9
Schelleman (2011)	+	+	+	+	+	-	5	+	+	-	-	+	3	8
Schelleman (2012)	+	+	+	+	+	-	5	+	+	+	-	+	4	9
Schelleman (2013)	+	+	+	+	+	-	5	+	+	+	-	+	4	9
Shin (2016)	+	+	+	+	+	+	6	+	+	+	+	+	5	11
Tadrous (2021)	+	+	+	+	+	+	6	+	+	+	+	+	5	11
Winterstein (2007)	+	+	+	-	+	+	5	+	+	+	+	-	4	9
Winterstein (2012)	+	+	+	+	+	+	6	-	+	+	+	-	3	9
Zhang (2015)	+	-	+	-	+	-	3	-	+	+	+	-	3	6

GRACE, Good Research for Comparative Effectiveness

© 2022 Zhang L et al. *JAMA Network Open.*

eFigure 1. Results of Leave-One-Out Sensitivity Analysis*

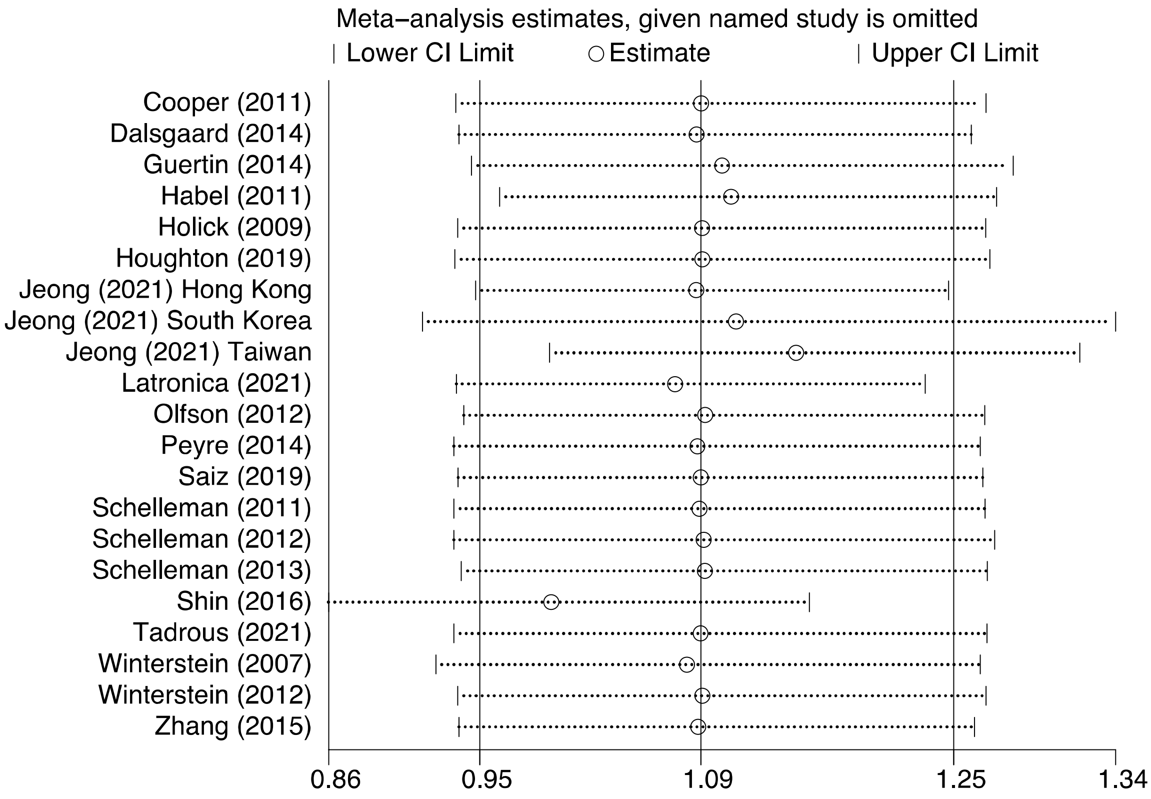

*The vertical axis shows the omitted study. Every circle indicates the pooled risk ratio when the left study is omitted in this meta-analysis. The two ends of every broken line represent the respective 95% confidence interval.

© 2022 Zhang L et al. *JAMA Network Open.*

eFigure 2. Results From Egger Test *

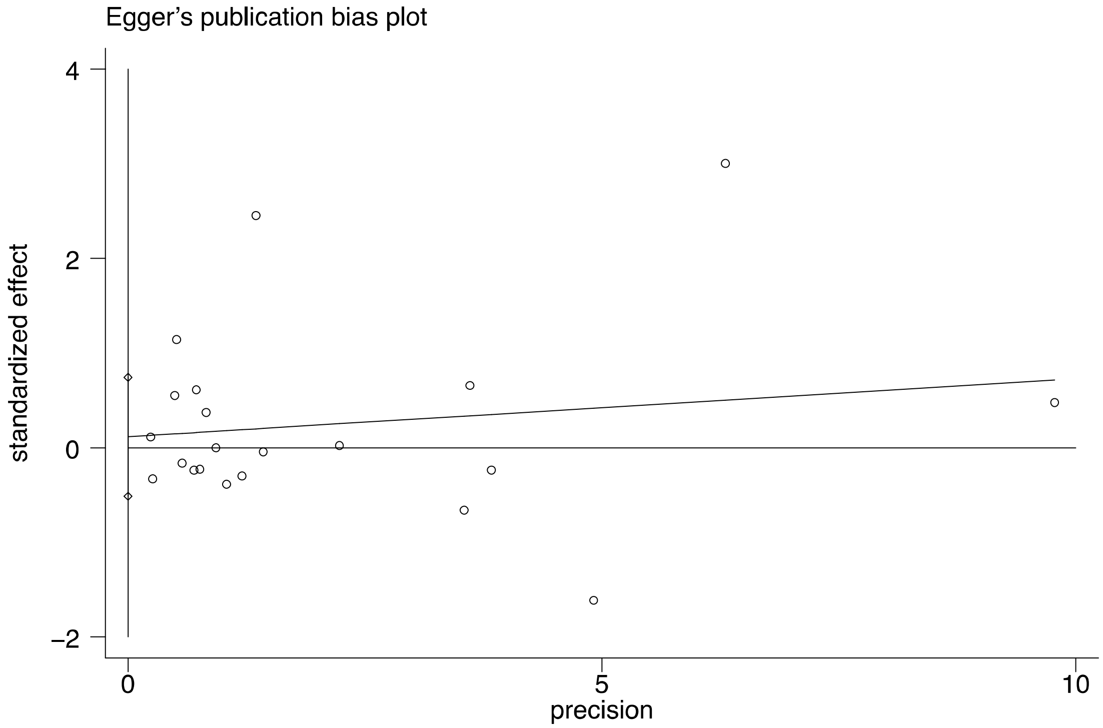

* Result from Egger's test for small study effect suggested there was no small study effects (P=0.71)

© 2022 Zhang L et al. *JAMA Network Open.*

eFigure 3. Publication Bias of Included Studies

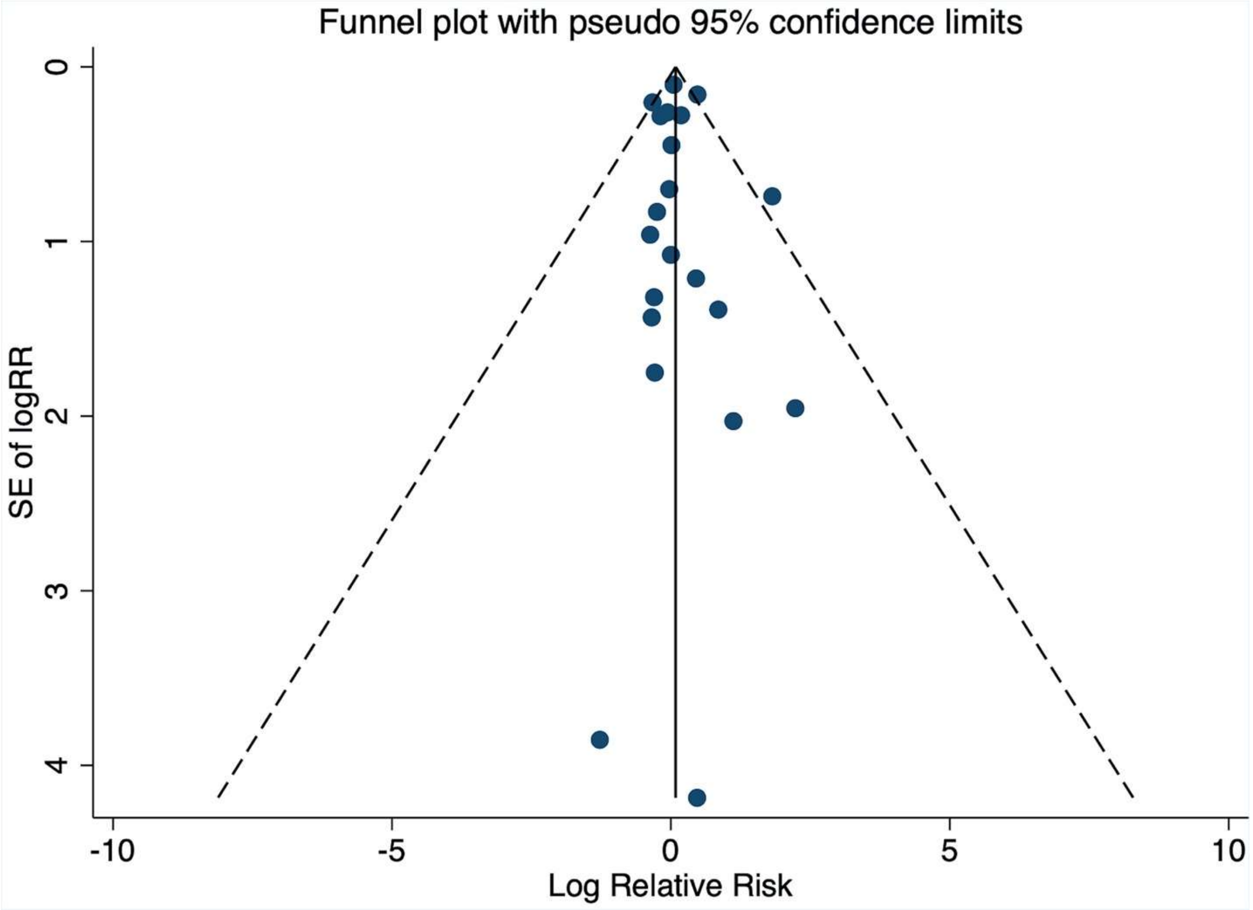

© 2022 Zhang L et al. *JAMA Network Open.*

eFigure 4. Associations of Stimulant and Nonstimulant ADHD Medication Use With CVD

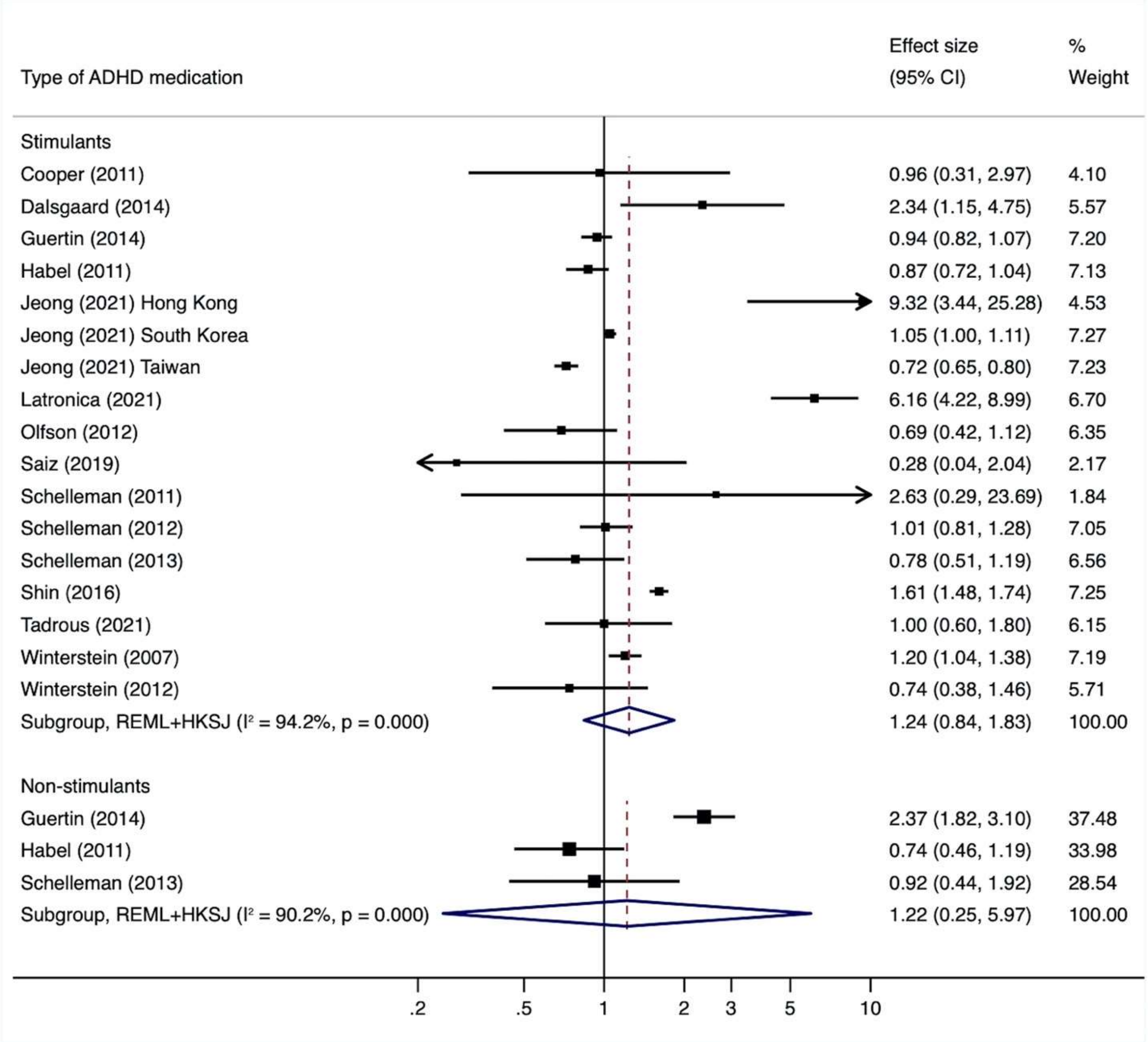

CVD, cardiovascular disease; REML, Restricted Maximum Likelihood; HKSJ, Hartung-Knapp-Sidik-Jonkman

© 2022 Zhang L et al. *JAMA Network Open.*

eFigure 5. Associations Between ADHD Medication Use and Specific CVD Outcomes

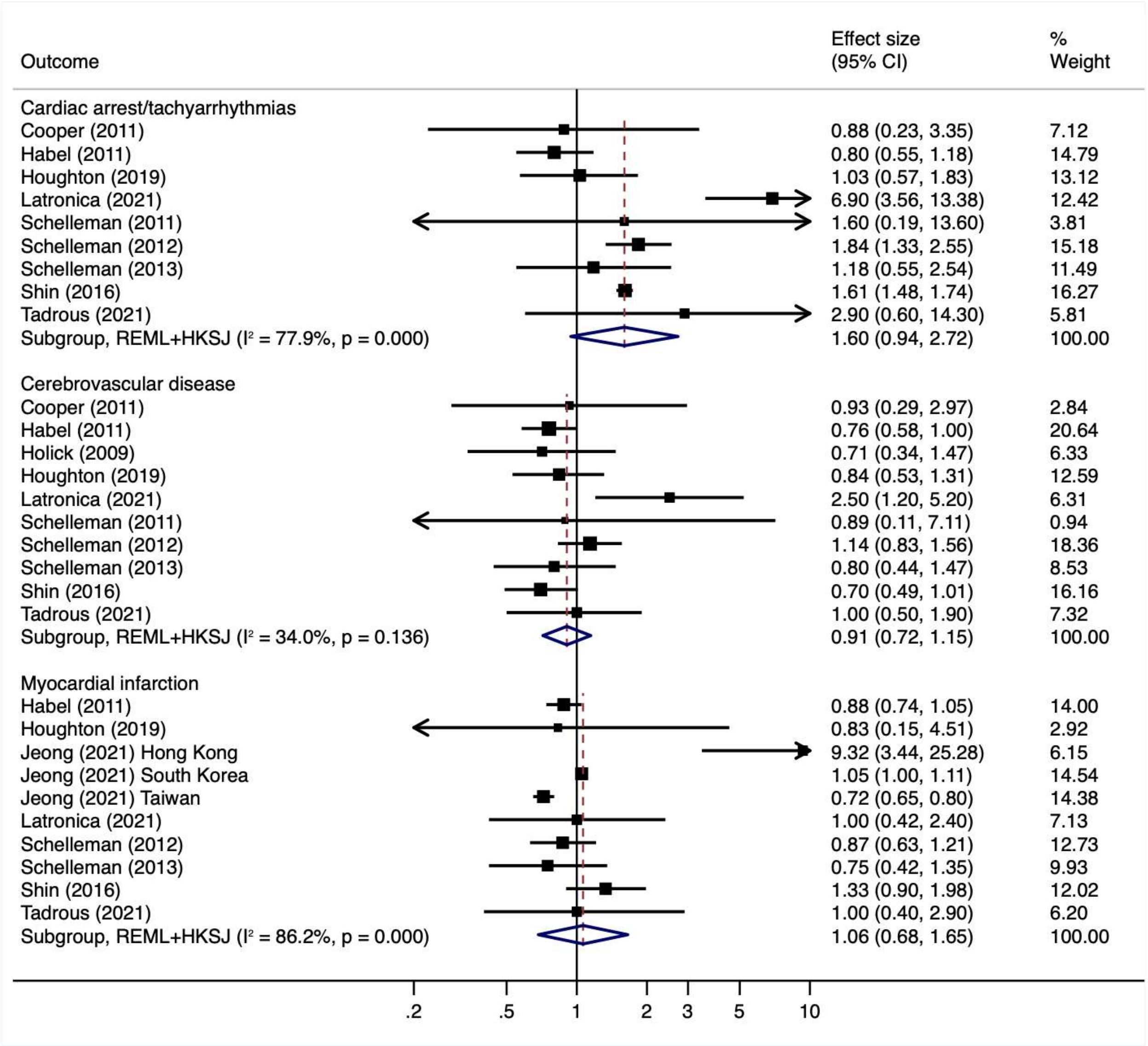

CVD, cardiovascular disease; REML, Restricted Maximum Likelihood; HKSJ, Hartung-Knapp-Sidik-Jonkman

© 2022 Zhang L et al. *JAMA Network Open.*

eFigure 6. Associations Between Stimulant ADHD Medication Use and Specific CVD Outcomes

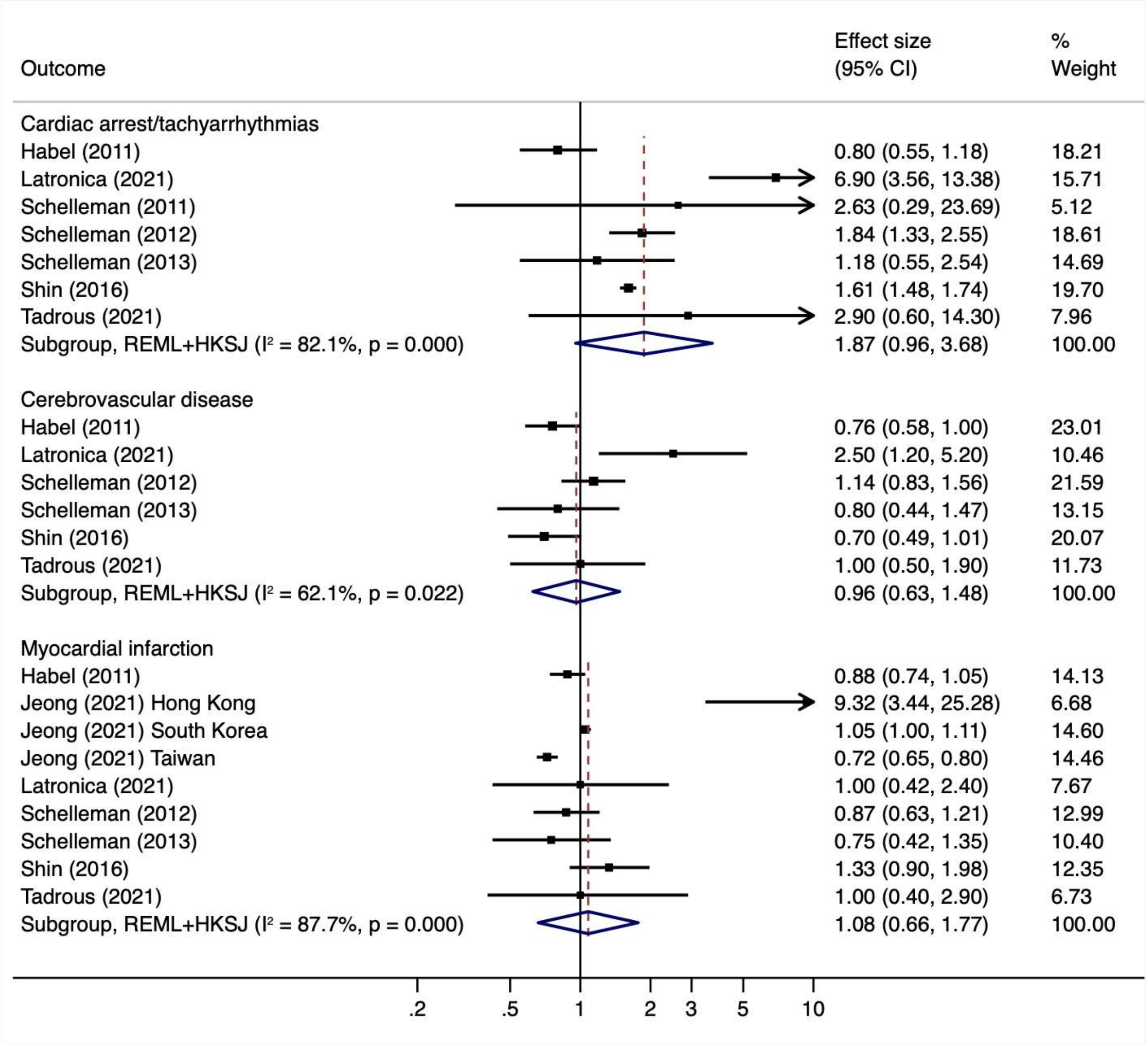

CVD, cardiovascular disease; REML, Restricted Maximum Likelihood; HKSJ, Hartung-Knapp-Sidik-Jonkman

© 2022 Zhang L et al. *JAMA Network Open.*

eFigure 7. Associations Between ADHD Medication Use and CVD, by Sex Group

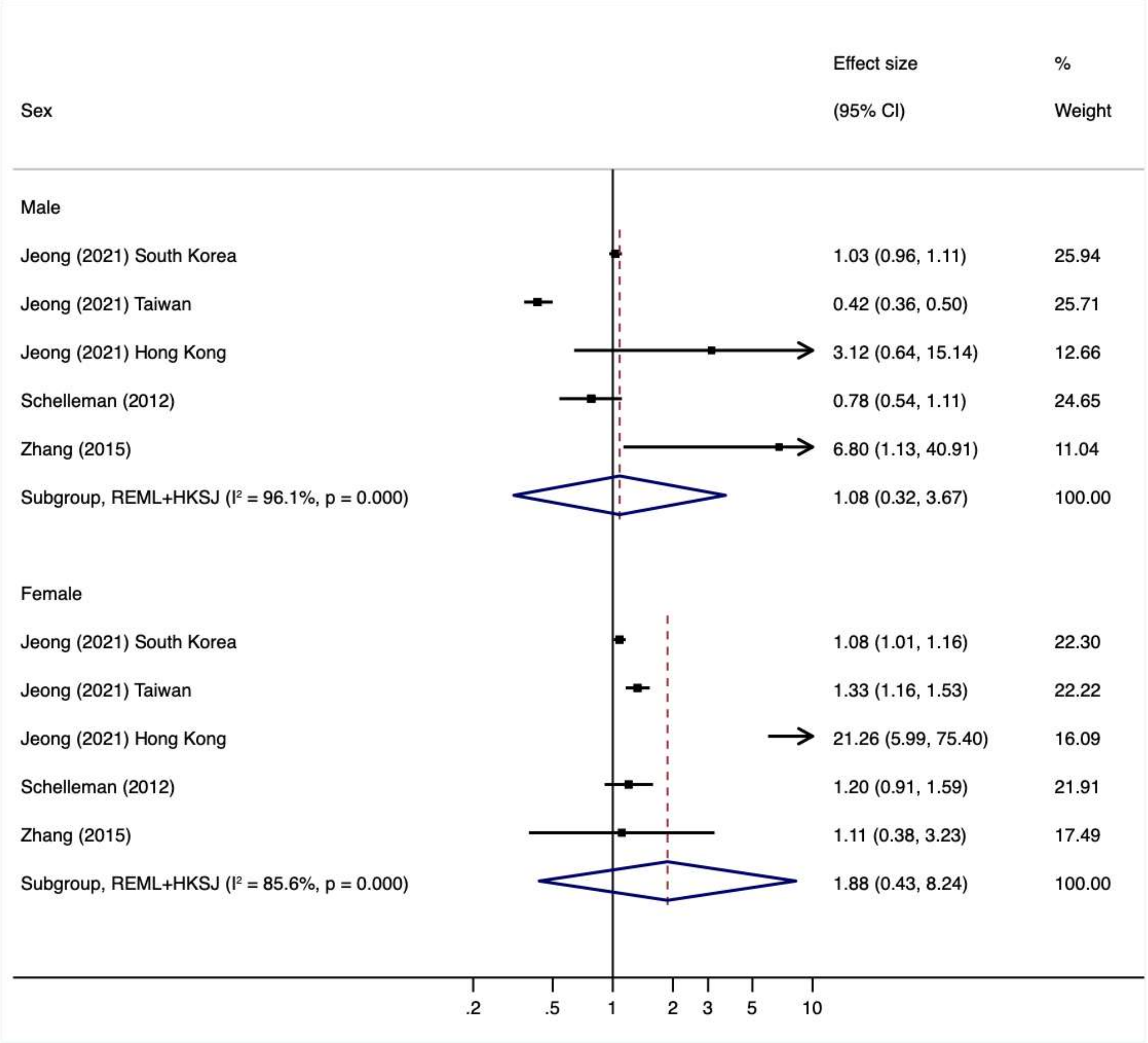

CVD, cardiovascular disease; REML, Restricted Maximum Likelihood; HKSJ, Hartung-Knapp-Sidik-Jonkman

eFigure 8. Associations Between ADHD Medication Use and CVD, by History of CVD

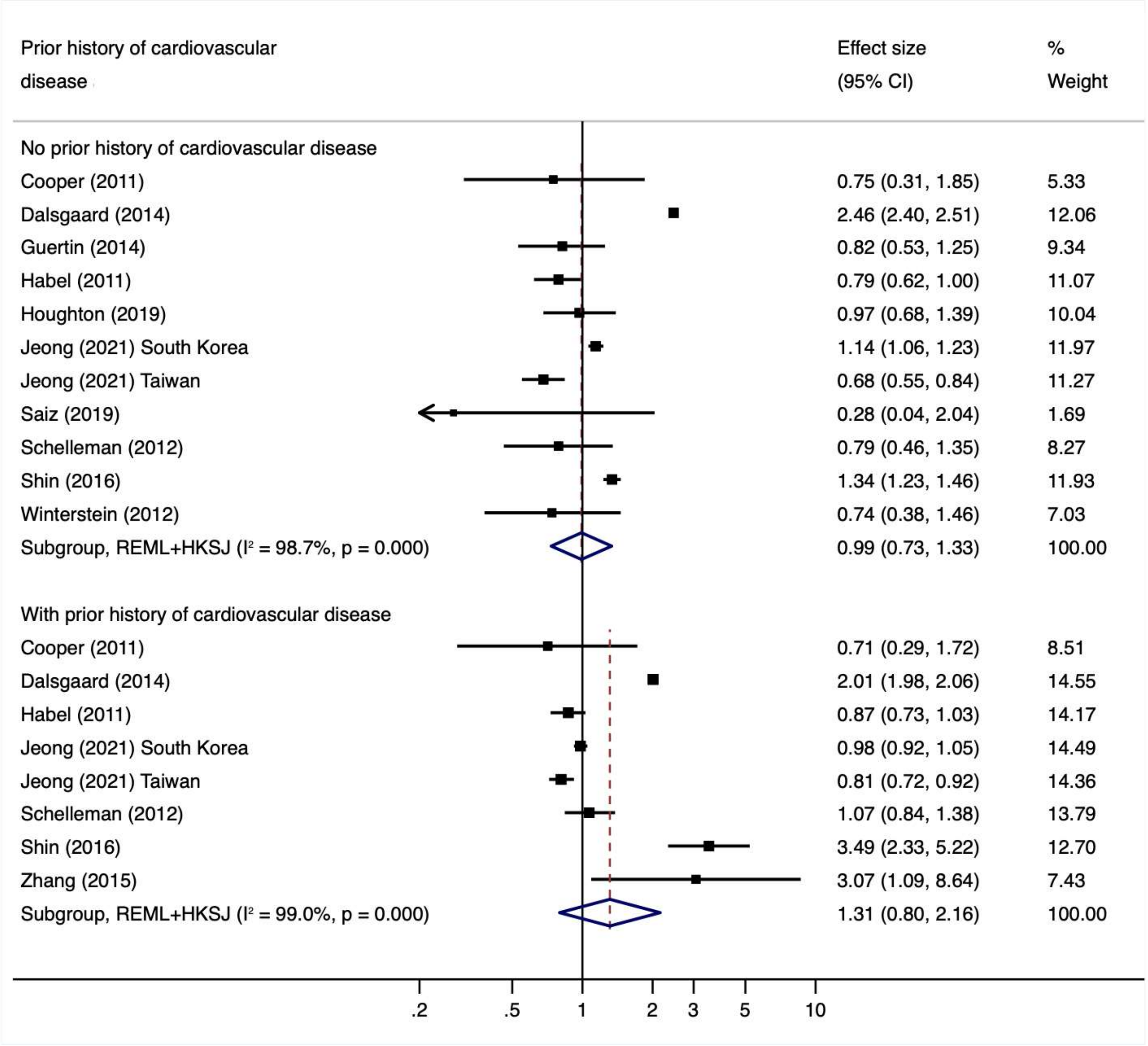

CVD, cardiovascular disease; REML, Restricted Maximum Likelihood; HKSJ, Hartung-Knapp-Sidik-Jonkman

© 2022 Zhang L et al. *JAMA Network Open.*

eReferences.

1. Habel LA, Cooper WO, Sox CM, et al. ADHD medications and risk of serious cardiovascular events in young and middle-aged adults. *JAMA: Journal of the American Medical Association.* 2011;306(24):2673-2683.
2. Holick CN, Turnbull BR, Jones ME, Chaudhry S, Bangs ME, Seeger JD. Atomoxetine and cerebrovascular outcomes in adults. *J Clin Psychopharmacol.* 2009;29(5):453-460.
3. Olfson M, Huang C, Gerhard T, et al. Stimulants and cardiovascular events in youth with attention-deficit/hyperactivity disorder. *Journal of the American Academy of Child & Adolescent Psychiatry.* 2012;51(2):147-156.
4. Schelleman H, Bilker WB, Kimmel SE, et al. Methylphenidate and risk of serious cardiovascular events in adults. *Am J Psychiatry.* 2012;169(2):178-185.
5. Schelleman H, Bilker WB, Kimmel SE, et al. Amphetamines, atomoxetine and the risk of serious cardiovascular events in adults. *PLoS One.* 2013;8(1):e52991.
6. Tadrous M, Shakeri A, Chu C, et al. Assessment of Stimulant Use and Cardiovascular Event Risks Among Older Adults. *JAMA Network Open.* 2021;4(10):e2130795-e2130795.
7. Winterstein AG, Gerhard T, Kubilis P, et al. Cardiovascular safety of central nervous system stimulants in children and adolescents: population based cohort study. *Bmj.* 2012;345:e4627.
8. Zhang C, Kutyifa V, Moss AJ, McNitt S, Zareba W, Kaufman ES. Long-QT Syndrome and Therapy for Attention Deficit/Hyperactivity Disorder. *J Cardiovasc Electrophysiol.* 2015;26(10):1039-1044.
9. Latronica JR, Clegg TJ, Tuan W-J, Bone C. Are Amphetamines Associated with Adverse Cardiovascular Events Among Elderly Individuals? *The Journal of the American Board of Family Medicine.* 2021;34(6):1074-1081.
10. Peyre H, Hoertel N, Hatteea H, Limosin F, Dubuc C, Delorme R. Adulthood self-reported cardiovascular risk and ADHD medications: results from the 2004-2005 National Epidemiologic Survey on Alcohol and Related Conditions. *J Clin Psychiatry.* 2014;75(2):181-182.
11. Winterstein AG, Gerhard T, Shuster J, Johnson M, Zito JM, Saidi A. Cardiac safety of central nervous system stimulants in children and adolescents with attention-deficit/hyperactivity disorder. *Pediatrics.* 2007;120(6):e1494-1501.
12. Cooper WO, Habel LA, Sox CM, et al. ADHD drugs and serious cardiovascular events in children and young adults. *The New England Journal of Medicine.* 2011;365(20):1896-1904.
13. Dalsgaard S, Kvist AP, Leckman JF, Nielsen HS, Simonsen M. Cardiovascular safety of stimulants in children with attention-deficit/hyperactivity disorder: a nationwide prospective cohort study. *Journal of child and adolescent psychopharmacology.* 2014;24(6):302-310.
14. Schelleman H, Bilker WB, Strom BL, et al. Cardiovascular events and death in children exposed and unexposed to ADHD agents. *Pediatrics.* 2011;127(6):1102-1110.
15. Guertin J, LeLorier J, Durand M, Gow R, Holbrook A, Levine M. Impact of a restrictive drug access program on the risk of cardiovascular encounters in children exposed to ADHD medications. *J Popul Ther Clin Pharmacol.* 2014;21(3):e357-369.

© 2022 Zhang L et al. *JAMA Network Open.*

16. Houghton R, de Vries F, Loss G. Psychostimulants/Atomoxetine and Serious Cardiovascular Events in Children with ADHD or Autism Spectrum Disorder. *Cns Drugs.* 2020;34(1):93-101.
17. Saiz LC, Gil M, Alonso A, Erviti J, Garjón J, Martínez M. Use of methylphenidate and risk for valvular heart disease: A case-control study nested in the BIFAP cohort. *Pharmacoepidemiol Drug Saf.* 2020;29(3):288-295.
18. Shin J-Y, Roughead E, Park B-J, Pratt N. Cardiovascular safety of methylphenidate among children and young people with attention-deficit/hyperactivity disorder (ADHD): Nationwide self controlled case series study. *BMJ.* 2016;353:i2550.
19. Jeong HE, Lee H, Lai EC-C, et al. Association between methylphenidate and risk of myocardial infarction: A multinational self-controlled case series study. *Pharmacoepidemiology and Drug Safety.* 2021;30(10):1458-1467.

© 2022 Zhang L et al. *JAMA Network Open.*
